# High-efficiency nonviral CRISPR/Cas9-mediated gene editing of human T cells using plasmid donor DNA

**DOI:** 10.1084/jem.20211530

**Published:** 2022-04-22

**Authors:** Soyoung A. Oh, Kate Senger, Shravan Madireddi, Ilseyar Akhmetzyanova, Isabel E. Ishizuka, Somayeh Tarighat, Jerry H. Lo, David Shaw, Benjamin Haley, Sascha Rutz

**Affiliations:** 1 Cancer Immunology, Genentech, South San Francisco, CA; 2 Molecular Biology, Genentech, South San Francisco, CA; 3 Cell Therapy Engineering and Development, Genentech, South San Francisco, CA; 4 Oncology Bioinformatics, Genentech, South San Francisco, CA

## Abstract

Genome engineering of T lymphocytes, the main effectors of antitumor adaptive immune responses, has the potential to uncover unique insights into their functions and enable the development of next-generation adoptive T cell therapies. Viral gene delivery into T cells, which is currently used to generate CAR T cells, has limitations in regard to targeting precision, cargo flexibility, and reagent production. Nonviral methods for effective CRISPR/Cas9-mediated gene knock-out in primary human T cells have been developed, but complementary techniques for nonviral gene knock-in can be cumbersome and inefficient. Here, we report a convenient and scalable nonviral method that allows precise gene edits and transgene integration in primary human T cells, using plasmid donor DNA template and Cas9-RNP. This method is highly efficient for single and multiplex gene manipulation, without compromising T cell function, and is thus valuable for use in basic and translational research.

## Teaser

Effective and precise gene editing of T lymphocytes is critical for advancing our understanding of T cell biology and the development of next-generation cellular therapies. The authors report a simple and efficient method for nonviral CRISPR/Cas9-based editing of T lymphocytes using plasmid donor DNA.

## Graphical Abstract

**Figure:**
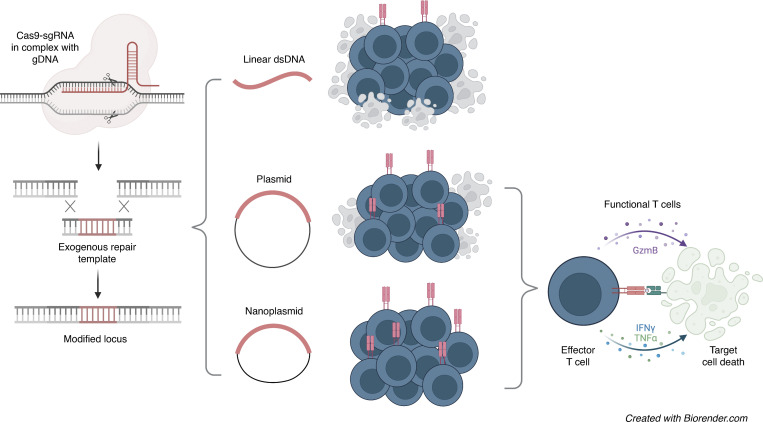

## Introduction

CRISPR-mediated gene knock-out using Cas9-ribonucleoprotein (RNP) delivery into primary human T cells represents a rapid and versatile approach for introducing genetic loss-of-function perturbations in this clinically relevant cell type (Schumann et al., 2015; Hendel et al., 2015; Seki and Rutz, 2018; Oh et al., 2019). However, methods for gain-of-function studies and stable expression of therapeutic transgenes in T cells rely mainly on viral delivery techniques that do not allow for the precise editing of genes.

Lentiviruses and γ-retroviruses are widely used by the research community and are also applied for the introduction of chimeric antigen receptors (CARs) and TCRs in the manufacturing of adoptive T cell therapies (Wang and Rivière, 2016; Zhang et al., 2017). Transposon-based gene delivery methods, such as the piggyBac and Sleeping Beauty systems, have been developed as nonviral alternatives (Monjezi et al., 2017; Kebriaei et al., 2016; Hudecek and Ivics, 2018). While these approaches yield high efficiencies of gene delivery, they are not amenable to precision gene editing and bear the risk of insertional mutagenesis, because the transgene is inserted into the host genome through random integration (Hacein-Bey-Abina et al., 2008, 2003; Modlich et al., 2009).

Homology-directed repair (HDR) of double-strand DNA breaks introduced by targeted gene editing methods, such as transcription activator–like effector nucleases, zinc finger nucleases, or CRISPR/Cas9, can be used to make precise changes to a genomic sequence, including the insertion of long stretches of DNA at a defined genomic location (Li et al., 2020; Singh et al., 2017). Viral vectors, in particular adeno-associated viruses (AAV), have been used to deliver donor DNA templates for HDR-mediated target gene knock-in in T cells (Sather et al., 2015; Wang et al., 2016; Eyquem et al., 2017; Choi et al., 2019). This approach was used, for instance, to insert a CAR construct into the T cell receptor α constant (*TRAC*) region locus, which placed the CAR under the control of the endogenous TCR promotor, thus improving its performance (Eyquem et al., 2017). Several studies have subsequently reported high editing efficiencies using AAV-based repair templates (Choi et al., 2019; Vakulskas et al., 2018; Dai et al., 2019). However, production and purification of AAV not only represents a significant clinical manufacturing challenge (Loo and van derWright, 2016; Halbert et al., 2018; Davidsson et al., 2020), it also limits more widespread use of this approach in the research community.

Recently, a series of seminal papers demonstrated that linear double-stranded DNA (dsDNA) donor templates can be co-delivered with Cas9-RNPs for directed insertion of full-length coding sequences at specific sites within the T cell genome (Nguyen et al., 2019; Roth et al., 2018; Schober et al., 2019), thus facilitating not only the generation of point mutants, but also the targeted integration of one or several expression constructs, including CARs or TCRs. Yet these methods require the production and purification of large quantities of linear dsDNA and yield only modest knock-in efficiencies, which constitute serious limitations to the utility and scalability of this approach. Here, we address these challenges by developing an efficient and scalable protocol for CRISPR/Cas9-mediated nonviral gene editing in primary human T cells using readily available plasmid-based donor templates.

## Results

### Plasmid-based DNA donors simplify CRISPR/Cas9-mediated gene knock-in in T cells and improve efficiency

Building on previous work, including a protocol for CRISPR/Cas9-mediated gene knock-out in human and murine T cells (Seki and Rutz, 2018; Oh et al., 2019) and a report describing the use of linear dsDNA as repair template (Roth et al., 2018), we set out to develop a robust, efficient, and scalable protocol for nonviral CRISPR/Cas9-mediated gene knock-in in primary human T cells. To circumvent the labor-intensive steps involved in the generation and purification of PCR-based linear dsDNA, and to facilitate T cell engineering with sequence-verified templates, we investigated the use of plasmid DNA. In addition to conventional plasmid backbones, which are ∼2.5 kb in size (i.e., pUC57), several smaller circularized DNA backbones including minicircles, midges, and nanoplasmids have been described for cell engineering applications (Hardee et al., 2017). Commercially available nanoplasmids consist of a <0.5-kb backbone (Luke et al., 2009; Williams et al., 2006). Since plasmid DNA is toxic to T cells (Mandal et al., 2014; Su et al., 2016), the use of these small backbone vectors could help to reduce the amount of DNA needed for transfection.

**Figure 1.**
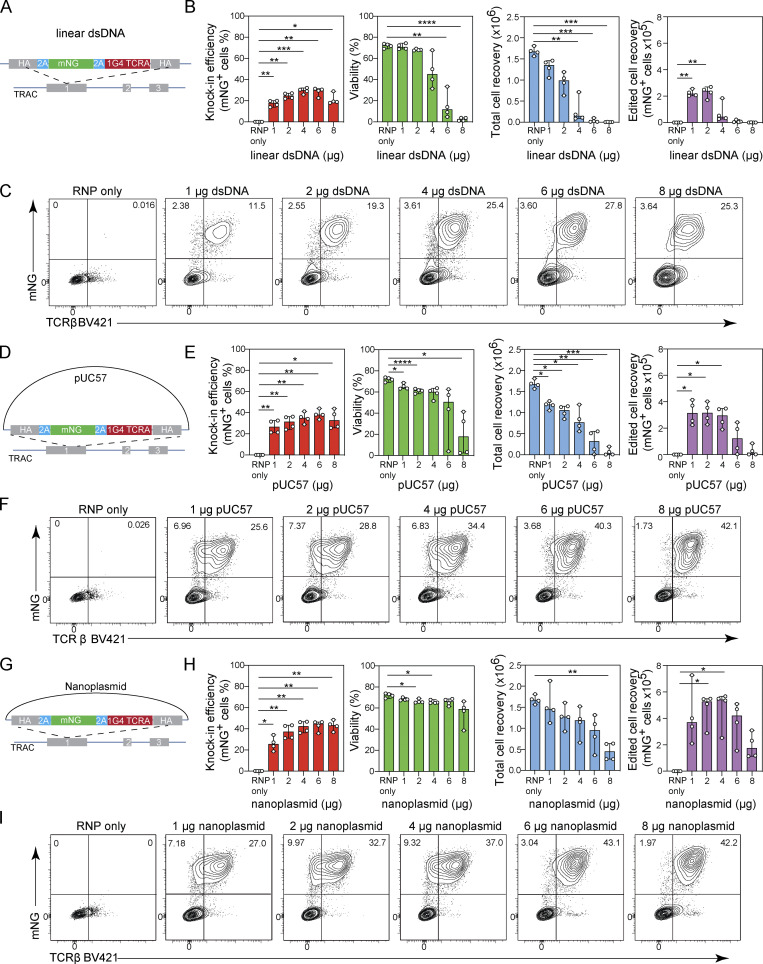
**Plasmid-based donor templates enable efficient nonviral gene editing of *TRAC* locus in primary T cells. (A–C)** Titration of linear dsDNA donor template. **(A)** Diagram of linear dsDNA knock-in construct *TRAC*-mNG. **(B)** Bar graphs depicting knock-in efficiency, cell viability, total cell recovery, and edited cell recovery (mNG-positive cells) 3 d after electroporation with 1, 2, 4, 6, or 8 µg of linear dsDNA donor template together with Cas9-RNP targeting the *TRAC* locus. Circles represent individual donors; bars represent median values with range (*n* = 4). **(C)** Representative contour plots showing the frequency of CD8^+^ T cells expressing mNG. **(D–F)** Titration of pUC57 plasmid donor template. **(D)** Diagram of pUC57 knock-in construct *TRAC*-mNG. **(E)** Bar graphs showing the frequency of CD8^+^ T cells expressing mNG, cell viability, total cell recovery, and edited cell recovery (mNG-positive cells) 3 d after electroporation with 1, 2, 4, 6, or 8 µg of pUC57 plasmid donor template together with Cas9-RNP targeting the *TRAC* locus. Circles represent individual donors; bars represent median values with range (*n* = 4). **(F)** Representative contour plots showing the frequency of CD8^+^ T cells expressing mNG. **(G–I)** Titration of nanoplasmid donor template. **(G)** Diagram of nanoplasmid knock-in construct *TRAC*-mNG. **(H)** Bar graphs showing the frequency of CD8^+^ T cells expressing mNG, total cell recovery, and edited cell recovery (mNG-positive cells) 3 d after electroporation with 1, 2, 4, 6, or 8 µg of nanoplasmid donor template together with Cas9-RNP targeting the *TRAC* locus. Circles represent individual donors; bars represent median values with range (*n* = 4). **(I)** Representative contour plots showing the frequency of CD8^+^ T cells expressing mNG. This experiment was performed twice. *, P < 0.05; **, P < 0.01; ***, P < 0.001; ****, P < 0.0001 in RM one-way ANOVA with Geisser–Greenhouse correction.

We designed a donor template with 500-bp homology arms targeting exon 1 of the *TRAC* locus and encoding the α chain of the NY-ESO-1–specific 1G4 TCR (Li et al., 2005) fused with the fluorescent protein mNeonGreen (mNG; Shaner et al., 2013). We generated this construct as linear dsDNA, as a pUC57 plasmid, or as a nanoplasmid (Fig. 1, A, D, and G). Similar to our previous approach to CRISPR/Cas9-mediated gene knock-out in T cells (Seki and Rutz, 2018), we optimized the process individually for CD8^+^ and CD4^+^ T cells, rather than working with a mixed cell population. Here, we first cultured isolated human CD8^+^ T cells in PRIME-XV media supplemented with the cytokines IL-7 and IL-15 and stimulated them with TransAct, a bead-free colloidal polymeric nanomatrix conjugated to humanized CD3 and CD28 agonists. The CD8^+^ T cells were cultured for 48 h before being nucleofected (4D nucleofection system; Lonza) with Cas9-RNPs containing a chemically-synthesized single guide RNA (sgRNA) targeting exon 1 of the *TRAC* locus together with the respective donor DNA template. All studies were performed using the R691A HiFi-Cas9 variant to minimize CRISPR/Cas9 off-target events (Vakulskas et al., 2018). Further, we titrated the amount of linear and plasmid DNA side-by-side and determined knock-in efficiency, cell viability, and cell recovery by flow cytometry 3 d after electroporation.

We found that 4 µg of linear dsDNA maximized the knock-in rate of 28.8–32.1% across four independent T cell donors (Fig. 1, B and C). However, this amount of dsDNA impaired cell viability and resulted in low T cell recovery (Fig. 1 B). When using 1 µg linear dsDNA instead, knock-in rates were lower (13.9–20%), but cell viability and recovery were improved and became comparable to the control condition, where transfection with Cas9-RNP was performed without dsDNA (Fig. 1, B and C). Titration of the pUC57 plasmid DNA revealed higher knock-in rates compared to linear dsDNA, with a rate of 33.5–44% when using as much as 6 µg of DNA (Fig. 1, E and F). However, this also resulted in compromised viability and cell recovery, whereas 2 µg plasmid DNA generated a 24–36.2% knock-in rate with minimally impaired cell viability, resulting in an optimal recovery of edited cells (Fig. 1, E and F). In contrast, the nanoplasmid format enabled high knock-in rates of 36.2–46.6% at 4 µg DNA, with minimal impact on cell viability and recovery compared with the control condition (Fig. 1, H and I), thus yielding nearly twice the number of edited cells compared with the pUC57 format or three times compared with linear dsDNA.

**Figure S1.**
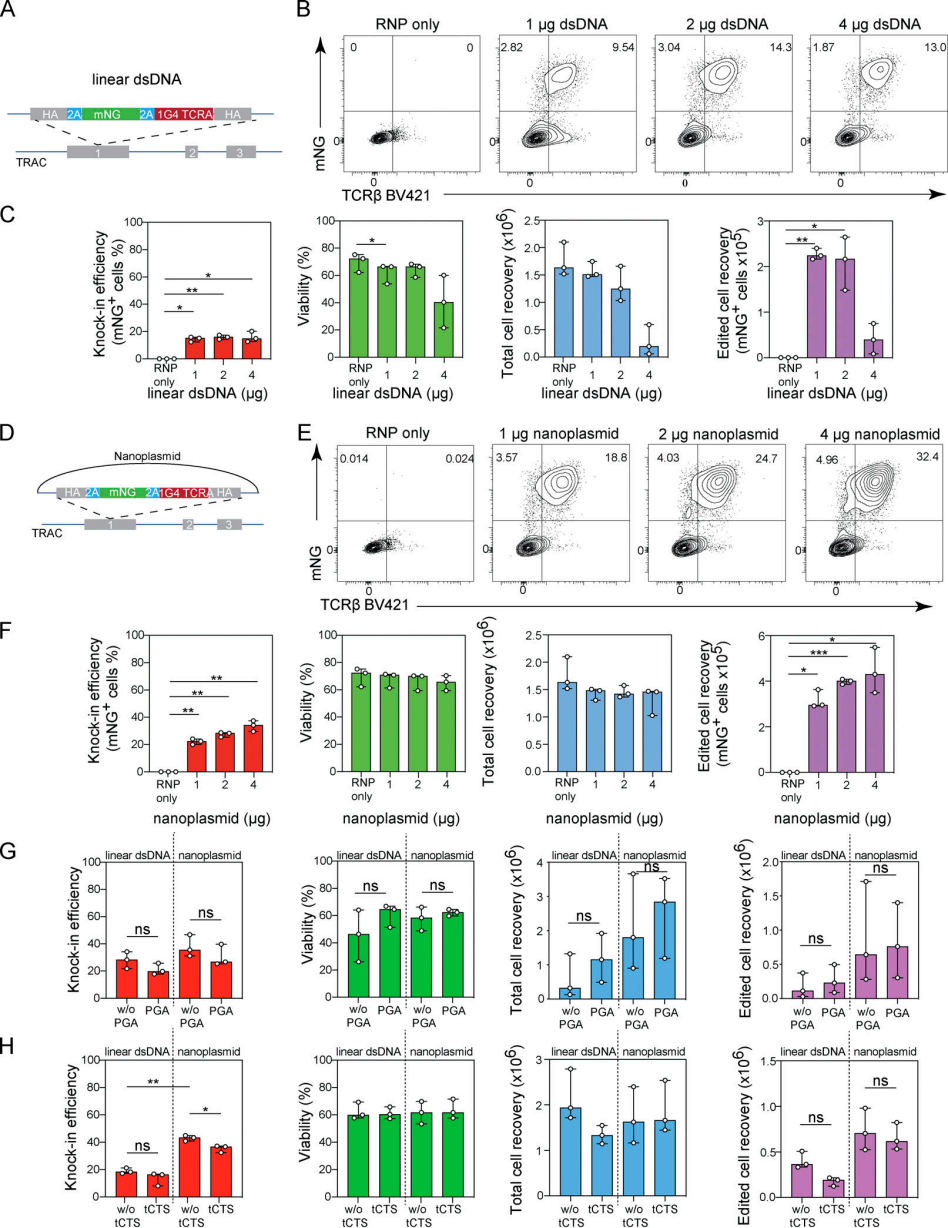
**Optimization of nonviral gene editing in primary T cells using plasmid-based donor templates. (A–F)** Titration of linear dsDNA and nanoplasmid donor templates in CD8^+ ^T cell cultures in RPMI/10% FBS medium. **(A)** Diagram of linear dsDNA knock-in construct *TRAC*-mNG. **(B)** Representative contour plots showing the frequency of CD8^+^ T cells expressing mNG. **(C)** Bar graphs depicting knock-in efficiency, cell viability, total cell recovery, and edited cell recovery (mNG-positive cells) of CD8^+^ T cells cultured in RPMI/10% FBS 3 d after electroporation with 1, 2, or 4 µg of linear dsDNA donor template together with Cas9-RNP targeting the *TRAC* locus. Circles represent individual donors; bars represent median values with range (*n* = 4). This experiment was performed twice. **(D)** Diagram of nanoplasmid knock-in construct *TRAC*-mNG. **(E)** Representative contour plots showing the frequency of CD8^+^ T cells expressing mNG. **(F)** Bar graphs depicting knock-in efficiency, cell viability, total cell recovery, and edited cell recovery (mNG-positive cells) of CD8^+^ T cells cultured in RPMI/10% FBS 3 d after electroporation with 1, 2, or 4 µg of nanoplasmid donor template together with Cas9-RNP targeting the *TRAC* locus. Circles represent individual donors; bars represent median values with range (*n* = 4). This experiment was performed twice. **(G)** Bar graphs depicting knock-in efficiency, cell viability, total cell recovery, and edited cell recovery 3 d after electroporation with 2 µg of either linear dsDNA or nanoplasmid donor template together with Cas9-RNP targeting the *TRAC* locus in the presence of absence of PGA. Circles represent individual donors; bars represent median values with range (*n* = 3). This experiment was performed twice. **(H)** Bar graphs depicting knock-in efficiency, cell viability, total cell recovery, and edited cell recovery 3 d after electroporation with 2 µg of either linear dsDNA or nanoplasmid donor template that either did or did not contain truncated Cas9 target sequences (tCTS) together with Cas9-RNP targeting the *TRAC* locus. Circles represent individual donors; bars represent median values with range (*n* = 3). This experiment has been performed twice. *, P < 0.05; **, P < 0.01; ***, P < 0.001 in RM one-way ANOVA with Geisser–Greenhouse correction (C and F) or paired *t* test (G and H).

We also evaluated the knock-in efficiency, cell viability, and recovery when CD8^+^ T cells were cultured in RPMI 1640 supplemented with 10% FBS (R10), which is a more commonly used culture medium for T cells. Experiments performed in R10 yielded results comparable to cultures in PRIME-XV media when using linear dsDNA templates but yield slightly lower knock-in rates (29.5–37.4%) when using nanoplasmid templates (Fig. S1, A–F). Yet, these results suggest that R10 may be used as an alternative to PRIME-XV media. Of note, addition of the negatively charged poly-L-glutamic acid (PGA) to the electroporation reaction, or encoding truncated Cas9 target sequences (tCTS) in the donor template (Nguyen et al., 2019), did not improve efficiency rates (Fig. S1, G and H).

**Figure S2.**
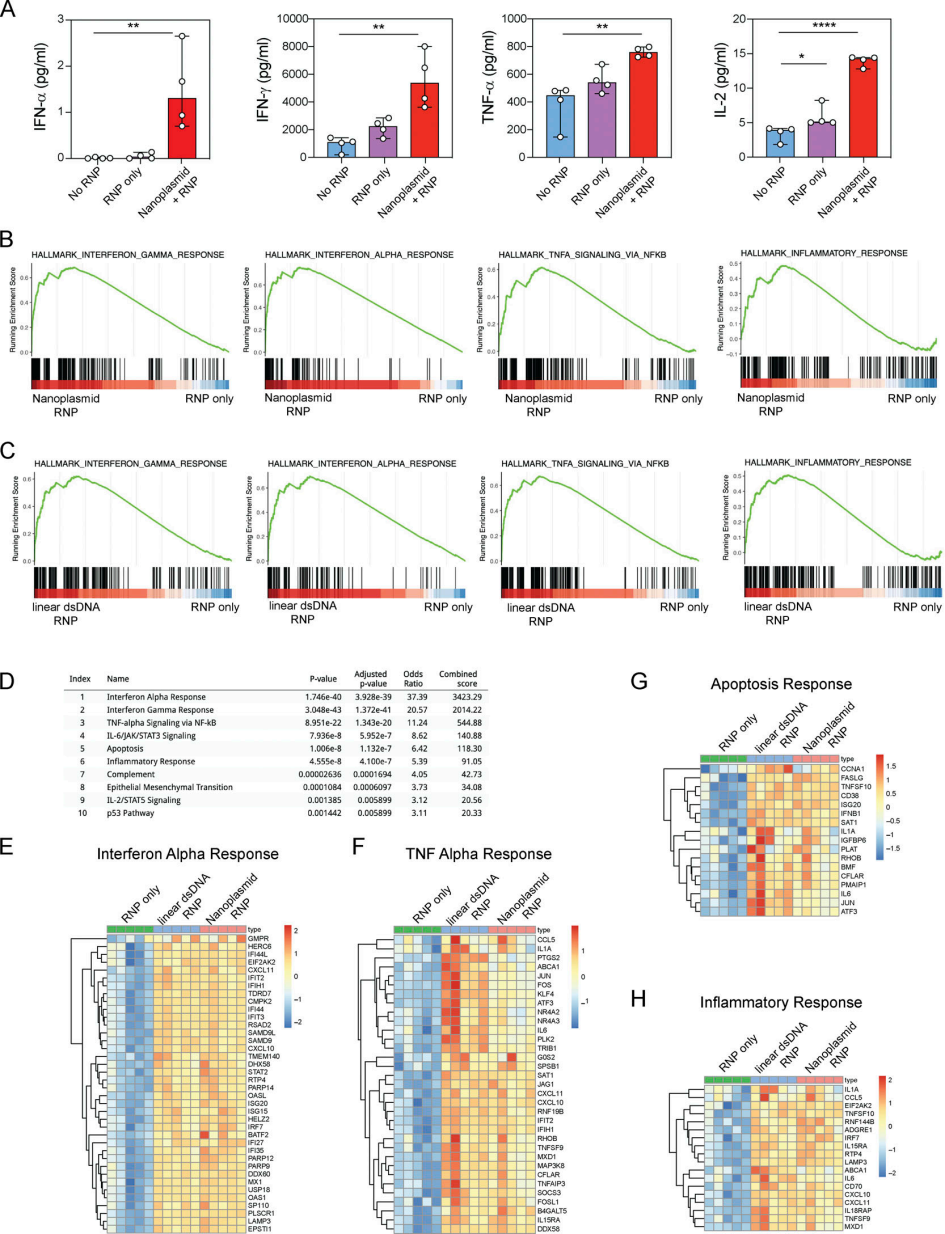
**Cytokine production and stress response induced in T cells following exposure to dsDNA donor templates. (A)** IFN-α measured by Simoa and IFN-γ, TNF-α, and IL-2 measured by Luminex from CD8^+^ T cells 18 h after transfection with Cas9-RNP targeting the *TRAC* locus alone or together with nanoplasmid donor template compared with non-transfected control T cells (No RNP). Circles represent individual donors; bars represent median values with range (*n* = 4). This experiment was performed once for Simoa and twice for Luminex. **(B)** GSEA from RNA-sequencing of CD8^+^ T cells after transfection with Cas9-RNP targeting the *TRAC* with nanoplasmid donor template compared with Cas9-RNP alone. Gene sets for IFN-γ response, IFN-α response, TNF-α signaling, and inflammatory response were significantly enriched. **(C)** GSEA from RNA-seq of CD8^+^ T cells after transfection with Cas9-RNP targeting the *TRAC* with linear dsDNA donor template compared to Cas9-RNP alone. Gene sets for IFN-γ response, IFN-α response, TNF-α signaling, and inflammatory response were significantly enriched. **(B and C)** The y axis represents enrichment score, and on the x axis are genes (vertical black lines) represented in gene sets. The colored band at the bottom represents the degree of differentially expressed genes (red for upregulation and blue for downregulation). **(D)** Gene set enrichment analysis of all 375 upregulated genes in both Nanoplasmid/Cas9-RNP and linear dsDNA/Cas9-RNP over Cas9-RNP-only using the GSEA MSigDB Hallmark 2020. **(E–H)** Heatmaps showing upregulated genes in Nanoplasmid/Cas9-RNP and linear dsDNA/Cas9-RNP over Cas9-RNP-only that mostly contributed to IFN-α response (E), TNF-α response (F), apoptosis (G), or inflammatory response (H; all MSigDB Hallmark). Color-coded by the normalized RNA-seq count data with variance stabilizing transformation (VST). This experiment was performed once. *, P < 0.05; **, P < 0.01; ****, P < 0.0001 in one-way ANOVA.

Our initial studies suggested that nanoplasmids had favorable qualities when used as a donor template in T cells, including improved knock-in efficiency and cell viability. However, plasmid DNA might induce stress responses in T cells, thus compromising cell viability or function. We therefore measured cytokine production after overnight culture of CD8^+^ T cells transfected with sg*TRAC* Cas9-RNP and nanoplasmid or Cas9-RNP alone. We also included unedited T cells as a control. Nanoplasmid transfection (but not Cas9-RNP alone) significantly induced IFN-α, IFN-γ, TNF-α, and IL-2 production (Fig. S2 A). RNA sequencing (RNA-seq) revealed up-regulation of gene expression signatures related to response to interferon-α, -β, and -γ as well as TNF-α in nanoplasmid transfected cells (Fig. S2 B). Importantly, linear dsDNA and nanoplasmid induced qualitatively and quantitatively similar stress-related responses (Fig. S2, C–H).

In conclusion, we found that plasmid DNA donor templates enabled highly efficient CRISPR/Cas9-mediated gene knock-in in CD8^+^ T cells, circumventing the need for linear dsDNA production and purification. While nanoplasmid vectors yielded generally more consistent results with higher knock-in rates, conventional plasmid backbones, such as pUC57, can be used successfully with careful titration.

### Optimization of CRISPR/Cas9-mediated gene knock-in with plasmid-based donor DNA in CD4^+^ and CD8^+^ T cells

**Figure 2.**
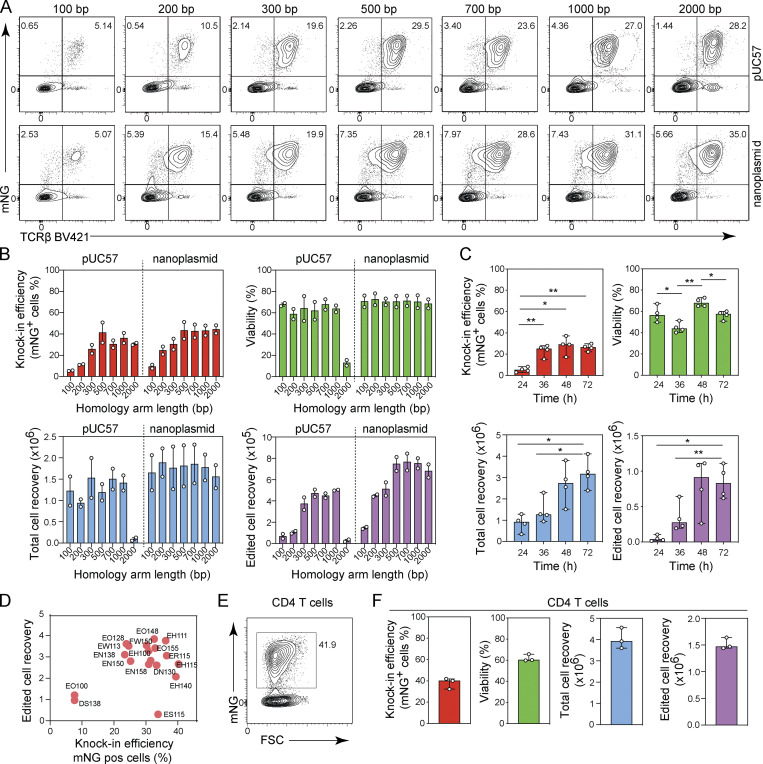
**Optimization of CRISPR/Cas9-mediated gene knock-in with plasmid-based donor DNA in CD4**^**+**^ **and CD8**^**+**^ **T cells. (A and B)** Homology arm optimization for plasmid-based donor templates. **(A)** Representative contour plots showing the frequency of CD8^+^ T cells expressing mNG. **(B)** Bar graphs depicting knock-in efficiency, cell viability, total cell recovery, and edited cell recovery (mNG-positive cells) 3 d after electroporation with pUC57 plasmid or nanoplasmid donor templates with homology arm lengths between 100 bp and 2,000 bp (amounts equimolar to 4 µg of the pUC57 2,000 bp construct) together with Cas9-RNP targeting the *TRAC* locus (*n* = 2). Circles represent individual donors; bars represent median values with range. This experiment was performed three times. **(C)** Frequency of CD8^+^ T cells expressing mNG, cell viability, total cell recovery, and edited cell recovery (mNG-positive cells) 3 d after electroporation after stimulating cells for 24, 36, 48, or 72 h prior to electroporation with nanoplasmid donor template together with Cas9-RNP targeting the *TRAC* locus (*n* = 4). Circles represent individual donors; bars represent median values with range. This experiment was performed twice. **(D)** Nucleofection pulse code optimization in CD8^+^ T cells electroporated with nanoplasmid donor template and Cas9-RNP targeting the *TRAC* locus. Graph shows frequency of cells expressing mNG and edited cell recovery (mNG-positive cells) 3 d after electroporation. Each circle represents a distinct pulse code. Data are representative of three independent CD8^+^ T cell donors. This experiment was performed twice. **(E and F)** Gene editing targeting the *TRAC* locus in CD4^+^ T cells. Representative contour plot showing the frequency of CD4^+^ T cells expressing mNG (E) and bar graphs (F) depicting knock-in efficiency, cell viability, total cell recovery, and edited cell recovery (mNG-positive cells) 5 d after electroporation of CD4^+^ T cells with *TRAC*-mNG nanoplasmid donor template together with Cas9-RNP targeting the *TRAC* locus (*n* = 3). Circles represent individual donors; bars represent median values with range. This experiment was performed twice. *, P < 0.05; **, P < 0.01 in RM one-way ANOVA with Geisser–Greenhouse correction.

We next compared plasmid donor templates with different homology arm lengths, ranging from 0.1 to 2 kb, using either pUC57 or nanoplasmid as backbone. The knock-in efficiency increased when using 0.1–0.5 kb homology arm lengths, irrespective of the backbone. A further extension did not change knock-in efficiency (Fig. 2, A and B). Importantly, cell viability and cell recovery were comparable with both backbones except when using the 2-kb homology arm pUC57 construct, which significantly impaired cell viability (Fig. 2, A and B). A homology arm length of 0.5 kb resulted in maximum knock-in efficiency and cell recovery (Fig. 2, A and B).

The timing of Cas9-RNP/nanoplasmid delivery following CD8^+^ T cell stimulation was also tested. We found that transfection 24 h after T cell stimulation resulted in significantly reduced knock-in efficiency when compared with transfection at later time points (Fig. 2 C). Cas9-RNP/nanoplasmid delivery at 48–72 h after stimulation resulted in maximal cell recovery (Fig. 2 C).

We further examined several nucleofection pulse codes to deliver Cas9-RNP and nanoplasmid templates and found that EH115 resulted in the highest target gene editing efficiency. Yet, other pulse codes such as EH111 increased cell recovery with minimal reduction in knock-in efficiency (Fig. 2 D). The selection of the nucleofection condition, therefore, should be done according to the priority given to either knock-in efficiency or cell recovery. For subsequent studies we used EH115.

Next, we cultured isolated human CD4^+^ T cells in PRIME-XV media supplemented with IL-2, IL-7, and IL-15. Cell stimulation was performed as described for CD8^+^ T cells, using TransAct for 48 h. We then nucleofected CD4^+^ T cells with *TRAC*-mNG nanoplasmid donor template and sg*TRAC* Cas9-RNP, and assessed the knock-in rate, cell viability, and recovery by flow cytometry. We observed a knock-in rate of 32.2–41.9% across three T cell donors, with viability and recovery rates similar to results observed with CD8^+^ T cells (Fig. 2, E and F). We concluded that this knock-in method was equally applicable to human CD4^+^ and CD8^+^ T cells.

### Efficient nonviral TCR editing using plasmid DNA donors

**Figure 3.**
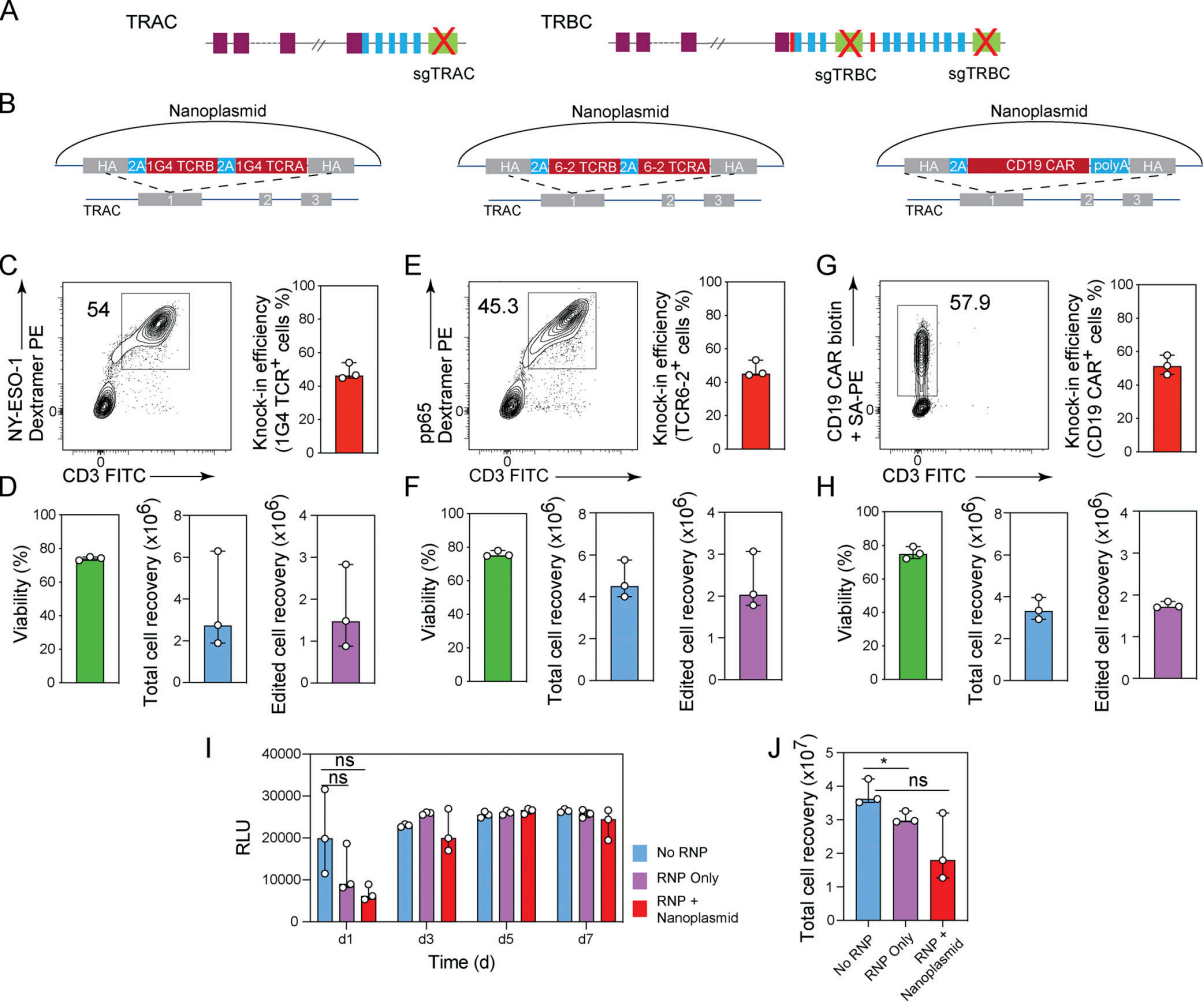
**Nonviral TCR editing using plasmid DNA donors. (A)** Diagram of TCR α and β genomic loci. V gene (purple), D gene (red), J gene (blue), and constant region (green) segments. sg*TRAC* and sg*TRBC* targeting sites are indicated. **(B)** Diagrams of nanoplasmid knock-in constructs *TRAC*-1G4TCR, *TRAC*-TCR6-2, and *TRAC*-CD19CAR. **(C, E, and G)** Representative contour plots (left) and bar graphs (right) showing the frequencies of CD8^+^ T cells expressing (C) a NY-ESO-1-specific 1G4 TCR, (E) a CMV-specific pp65 6-2 TCR, and (G) a CD19-CAR 5 d after electroporation using nanoplasmid donor templates together with Cas9-RNPs targeting the *TRAC* locus. **(D, F, and H)** Bar graphs showing the cell viability, total cell recovery, and edited cell recovery 5 d after electroporation using nanoplasmid donor templates encoding (D) a NY-ESO-1–specific 1G4 TCR, (F) a CMV-specific pp65 6-2 TCR, and (H) a CD19-CAR together with Cas9-RNPs targeting the *TRAC* locus. Circles represent individual donors; bars represent median values with range (*n* = 3). This experiment was performed three times. **(I)** Lactate levels in culture supernatant analyzed by luminescence using the Lactate-Glo Assay were measured 1, 3, 5, and 7 d after transfection of CD8^+^ T cells with sg*TRAC*/sg*TRBC* Cas9-RNP (RNP only) or sg*TRAC*/sg*TRBC* Cas9-RNP and nanoplasmid donor template targeting the *TRAC* locus (RNP + nanoplasmid) compared with non-transfected control T cells (No RNP); RLU, relative light units. **(J)** Number of cells recovered from cultures 7 d after transfection of CD8^+^ T cells with sg*TRAC*/sg*TRBC* Cas9-RNP (RNP only) or sg*TRAC*/sg*TRBC* Cas9-RNP and nanoplasmid donor template targeting the *TRAC* locus (RNP + nanoplasmid) compared with non-transfected control T cells (No RNP). This experiment was performed three times. *, P < 0.05 in RM one-way ANOVA with Geisser–Greenhouse correction.

**Figure S3.**
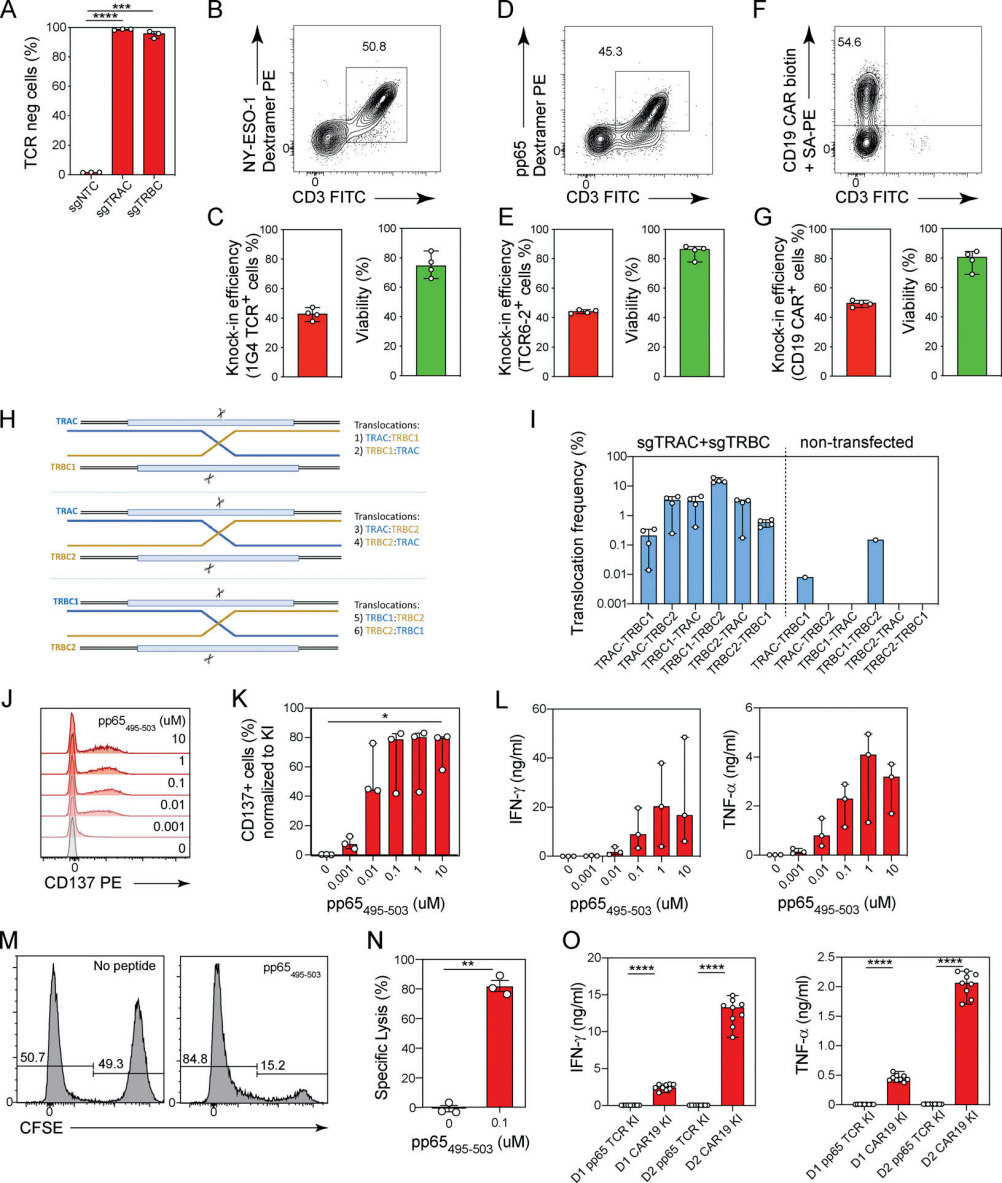
**Nonviral TCR editing in CD4**^**+**^ **and CD8**^**+**^ **T cells using plasmid DNA donors. (A)** TCR expression on the cell surface by flow cytometry of CD8^+^ T cells 48 h after transfection with Cas9-RNP targeting the *TRAC* (sg*TRAC*) or *TRBC* (sg*TRBC*) loci. Circles represent individual donors; bars represent median values with range (*n* = 3). This experiment was performed three times. **(B–G)** TCR editing in CD4^+^ T cells. Representative contour plots showing the frequencies of CD4^+^ T cells expressing a NY-ESO-1-specific 1G4 TCR (B), a CMV-specific pp65 6-2 TCR (D), and a CD19-CAR (F) and bar graphs showing the knock-in efficiency and cell viability 5 d after electroporation using nanoplasmid donor templates encoding a NY-ESO-1-specific 1G4 TCR (C), a CMV-specific pp65 6-2 TCR (E), and a CD19-CAR (G) together with Cas9-RNPs targeting the *TRAC* locus. Circles represent individual donors; bars represent median values with range (*n* = 4). This experiment was performed twice. **(H and I)** Diagram depicting all possible translocation events between the *TRAC*, *TRBC1*, and *TRBC2* genomic loci (H). Bar graph (I) showing the frequencies of individual translocation events between the *TRAC*, *TRBC1*, and *TRBC2* genomic loci quantified by ddPCR in CD8^+^ T cells co-transfected with Cas9-RNPs targeting the *TRAC* and *TRBC* loci or in non-transfected control T cells. Circles represent individual donors; bars represent median values with range (*n* = 4). This experiment was performed twice. **(J and K)** Representative histograms (J) and bar graphs (K) showing proportions of CD137-expressing pp65 TCR knock-in CD8^+^ T cells stimulated with indicated concentrations of pp65_495–503_ peptide. Circles represent individual donors; bars represent median values with range (*n* = 3). This experiment was performed twice. **(L)** Bar graphs showing IFN-γ and TNF-α production by pp65 TCR knock-in CD8^+^ T cells stimulated with indicated concentrations of pp65_495–503_ peptide. Circles represent individual donors; bars represent median values with range (*n* = 3). This experiment was performed twice. **(M)** Representative histograms showing the frequencies of CFSE-positive target cells and CFSE-negative reference cells in co-cultures with pp65 TCR knock-in CD8^+^ T cells in the absence or presence of the cognate peptide. **(N)** Graphs showing specific lysis calculated in the absence of peptide or with 0.1 µM of pp65_495–503_ peptide. Circles represent individual donors; bars represent median values with range (*n* = 3). This experiment was performed twice. **(O)** Bar graphs showing IFN-γ and TNF-α production by TCR6-2 (irrelevant TCR) or CD19-CAR knock-in CD4^+^ T cells from two donors (D1 and D2) in co-cultures with CD19-expressing B cells. Circles represent technical replicates; bars represent median values with range (*n* = 9). This experiment was performed twice. *, P < 0.05; **, P < 0.01; ***, P < 0.001; ****, P < 0.0001 in RM one-way ANOVA with Geisser–Greenhouse correction (A and K), paired *t* test (N), and one-way ANOVA (O).

We next applied our method to attempt TCR editing in human T cells. The knock-in of an engineered TCR with a desired antigen specificity requires the knock-out of the endogenous TCR in order to prevent mis-pairing with its α and β chains. By targeting the transgenic TCRs to the *TRAC* locus, the endogenous TCR α chain is disrupted, resulting in expression of a truncated form. However, the TCR β chain needs to be knocked-out separately. We therefore used a sg*TRBC* guide that targets exon 1 of both the *TRBC1* and *TRBC2* loci (Fig. 3 A), leading to complete loss of TCR expression as detected by flow cytometry (Fig. S3 A). We next designed donor templates encoding TCRs with known specificity: the NY-ESO-1–specific 1G4 TCR and the CMV A2/pp65_495–503_-specific TCR6-2 (Schober et al., 2019), as well as a human CD19-specific CAR (Bloemberg et al., 2020), all targeting the *TRAC* locus (Fig. 3 B).

We stimulated and cultured CD8^+^ T cells for 48 h as before, and co-transfected with sg*TRAC* and sg*TRBC*-containing Cas9-RNPs, together with 4 µg of TCR or CAR-encoding nanoplasmids and assessed TCR expression 5 d later by flow cytometry. We detected 1G4 TCR expression on the surface of 44.9–54% of T cells with minimal impact on cell viability (Fig. 3, C and D). Starting with 2 × 10^6^ CD8^+^ T cells, we recovered between 0.88–2.88 × 10^6^ 1G4 TCR positive T cells 5 d after electroporation (Fig. 3 D). T cells negative for 1G4 expression did not express endogenous TCR complexes on their surface, demonstrating the highly efficient gene knock-out (Fig. 3 C). Transfections with nanoplasmids encoding TCR6-2 or CD19 CAR constructs yielded similar results, with 44.4–53.3% and 46.3–57.9% knock-in rates (Fig. 3, E and G), respectively, and cell recovery of 1.7–3.1 × 10^6^ edited cells (Fig. 3, F and H). Again, the endogenous TCR was knocked out in virtually all T cells (Fig. 3, E and G).

When we performed TCR editing in isolated CD4^+^ T cells using the same targeting strategy and TCR or CAR donor templates, we observed knock-out and knock-in rates comparable to our results with CD8^+^ T cells (Fig. S3, B–G).

When attempting multiplexed gene editing, such as the simultaneous *TRAC* and *TRBC* knock-in/knock-out approach used here, the occurrence of chromosomal translocations between the cut sites has to be considered. We designed a digital-droplet (dd)PCR assay to quantify all possible translocation events involving the *TRAC*, *TRBC1*, and *TRBC2* loci (Fig. S3 H). While translocations between the *TRAC* and *TRBC1* or *TRBC2* loci occurred with frequencies of 0.01–4.4% depending on the orientation of the translocation and the donor, fusions of the neighboring *TRBC1* and *TRBC2* loci (corresponding to the deletion of 9.3 kb) occurred with frequencies of 13.1–19% (Fig. S3 I). These numbers were in line with a previous report (Stadtmauer et al., 2020).

We next determined how the gene editing process affected the expansion of T cells in culture. For this, we used the G-Rex culture system that allows high cell densities and simple medium exchanges without the need for splitting or replating of cells over the course of 1 wk. To minimize disturbing the cell cultures, we measured lactate levels over time, as a proxy for cell metabolism and culture performance. Cell recovery was quantified at the end of the study. Our data demonstrated that cell growth and metabolic activity were impaired on day 1 after transfection, when Cas9-RNPs were used either alone and more so when Cas9-RNPs were used together with nanoplasmid donor template compared to non-edited cells (Fig. 3 I). However, in both cases cultures recovered by day 3 after transfection, and grew similarly to control cells throughout the remaining time in culture. Cell recovery on day 7 was similar between control (unedited) T cells and the Cas9-RNP-only (knock-out) condition, yielding 3.5–4.4 × 10^7^ and 2.9–3.2 × 10^7^ cells, respectively. Cultures transfected with Cas9-RNP/nanoplasmid (knock-out/knock-in) in comparison yielded 1.3–3.3 × 10^7^ T cells, with a median of 1.8 × 10^7^ cells or about half of the cells in the unedited control (Fig. 3 J).

These data suggested that our method enabled highly efficient TCR editing, with near complete knock-out of the endogenous TCR and knock-in of the transgenic TCR in up to 60% of T cells with minimal impact on viability and growth kinetics of the cells.

### TCR-engineered T cells recognize and kill antigen-expressing target cells

**Figure 4.**
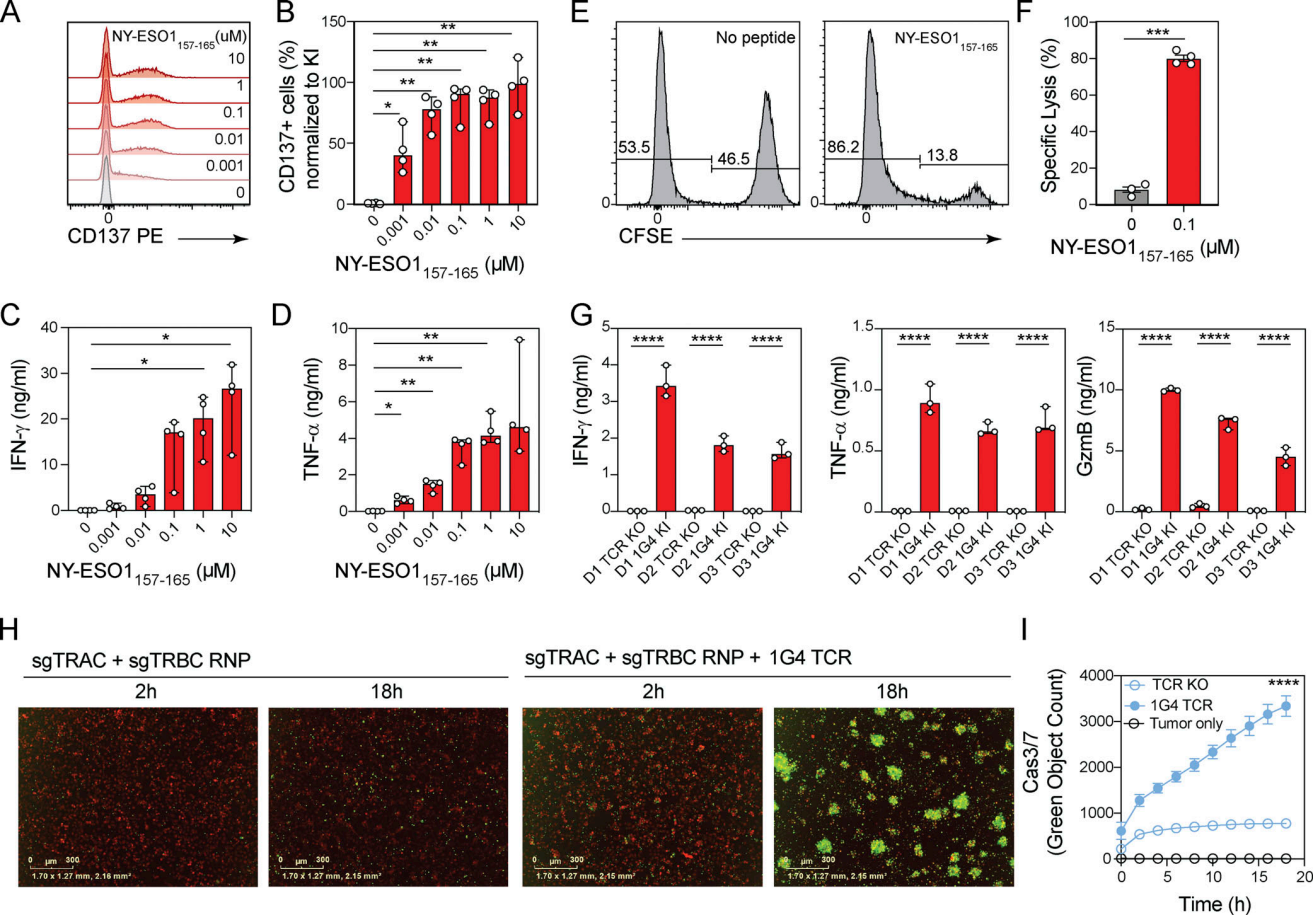
**TCR-engineered T cells recognize and kill antigen-expressing target cells. (A and B)** Representative histograms (A) and bar graphs (B) showing proportion of CD137 expression of 1G4 TCR knock-in CD8^+^ T cells stimulated with indicated concentrations of NY-ESO-1_157–165_ peptide. Circles represent individual donors; bars represent median values with range (*n* = 4). This experiment was performed twice. **(C and D)** Bar graphs showing IFN-γ (C) or TNF-α (D) production by 1G4 TCR knock-in CD8^+^ T cells stimulated with indicated concentrations of NY-ESO-1_157–165_ peptide. Circles represent individual donors; bars represent median values with range (*n* = 4). This experiment was performed twice. **(E)** Representative histograms showing the frequencies of CFSE-positive target cells and CFSE-negative reference cells in co-cultures with 1G4 TCR knock-in CD8^+^ T cells in the absence or presence of the cognate peptide. **(F)** Graphs showing specific lysis calculated in the absence of peptide or with 0.1 µM of NY-ESO-1_157–165_ peptide. Circles represent individual donors; bars represent median values with range (*n* = 4). This experiment was performed twice. **(G)** Bar graphs showing IFN-γ, TNF-α, and granzyme B (GzmB) production by TCR knock-out or 1G4 TCR knock-in CD8^+^ T cells from three donors co-cultured with A-375 cells that express the NY-ESO-1 antigen. Circles represent technical replicates; bars represent median values with range (*n* = 3). This experiment was performed twice. **(H)** Representative images for A-375 cells that express the NY-ESO-1 antigen and were labeled with a cytoplasmic dye and co-cultured with TCR knock-out CD8^+^ T cells (left) or 1G4 TCR knock-in CD8^+^ T cells (right) 2 and 18 h after culture seeding in the presence of caspase 3/7-green apoptosis reagent. Scale bars indicate 300 µm distance. **(I)** Representative target cell killing over time as measured by the Cas3/7-positive object count in co-cultures of A-375 cells expressing the NY-ESO-1 antigen and labeled with a cytoplasmic dye and co-cultured with TCR knock-out CD8^+^ T cells (open circles) or 1G4 TCR knock-in CD8^+^ T cells (filled circles). Mean values ± SD of six technical replicates. This experiment was performed twice with three independent donors per experiment. *, P < 0.05; **, P < 0.01; ***, P < 0.001; ****, P < 0.0001 in RM one-way ANOVA with Geisser–Greenhouse correction (B–D); paired *t* test (F); one-way ANOVA (G); or Tukey’s multiple comparisons test, two-way ANOVA (I).

Having demonstrated efficient TCR knock-in, we next assessed whether these engineered T cells were functional and able to recognize antigen-expressing target cells. To this end, we harvested T cells engineered to express either the NY-ESO-1–reactive 1G4 TCR or CMV A2/pp65_495–503_ TCR6-2 on day 8 of culture. T cells were then co-cultured overnight with a HLA-A02:01 positive B cell line pulsed with increasing concentrations of NY-ESO-1_157–165_ or pp65_495–503_ peptide at a 1:1 effector-to-target-cell (E:T) ratio. No T cell activation was observed in the absence of exogenously added peptide, suggesting that the removal of endogenous TCRs effectively prevented any alloreactivities (Fig. 4, A–F; and Fig. S3, J–N). The engineered T cells up-regulated CD137 expression in a peptide concentration-dependent manner (Fig. 4, A and B; and Fig. S3, J and K). Similarly, levels of IFN-γ and TNF-α in co-culture supernatants increased with peptide concentration (Fig. 4, C and D; and Fig. S3 L). To test antigen-specific target cell killing, we co-cultured 1G4 or TCR6-2 TCR-engineered T cells with peptide-pulsed CSFE-labeled B cells. To quantify specific target cell lysis, we mixed at a 1:1 ratio CFSE-labeled cells pulsed with 0.1 µM cognate peptide and non-labeled control B cells and measured the ratio of CFSE-positive to CFSE-negative cells by flow cytometry after overnight co-culture with TCR-engineered T cells. At an E:T ratio of 1:1, we observed 77–81% and 76–89% specific target cell lysis for the NY-ESO-1 and pp65 peptide, respectively (Fig. 4, E and F; and Fig. S3, M and N), thus demonstrating the highly potent cytotoxic potential of our TCR-edited cells.

We next tested T cell activation and cytotoxic activity against target cells with endogenous antigen expression. A-375 cells, which express the NY-ESO-1 antigen, were co-cultured with 1G4 TCR-expressing or TCR knock-out T cells at a 1:1 E:T ratio. Supernatants of co-cultures with 1G4 TCR-engineered T cells, but not those with TCR knock-out T cells, contained IFN-γ, TNF-α, and granzyme B after 18 h (Fig. 4 G). To monitor target cell lysis, we had labeled A-375 cells in these co-cultures with Incucyte Nuclight Rapid Red Dye. Target cell lysis over time was quantified using the caspase 3/7 green apoptosis reagent. We detected rapid and potent target cell lysis over the course of 18 h in the presence of 1G4 TCR-expressing T cells, but not in control cultures (Fig. 4, H and I).

We next engineered CD4^+^ T cells to express either a CD19-specific CAR or the pp65-specific TCR6-2 and co-cultured them for 24 h with CD19-expressing Granta-519 B cells at an E:T ratio of 1:1. CD19 CAR-expressing T cells, but not those carrying TCR6-2, whose cognate antigen is not expressed by Granta-519 B cells, produced IFN-γ and TNF-α, demonstrating CAR-dependent CD4^+^ T cell activation (Fig. S3 O).

Thus, T cells engineered to express TCRs or CARs using our plasmid-based CRISPR/Cas9-mediated gene editing approach were functional, as demonstrated by potent antigen-dependent T cell activation, cytokine production, and cytolytic activity at a low E:T cell ratio.

### Promoter-containing nanoplasmids enable targeted gene knock-in and prolonged transient gene expression

**Figure S4.**
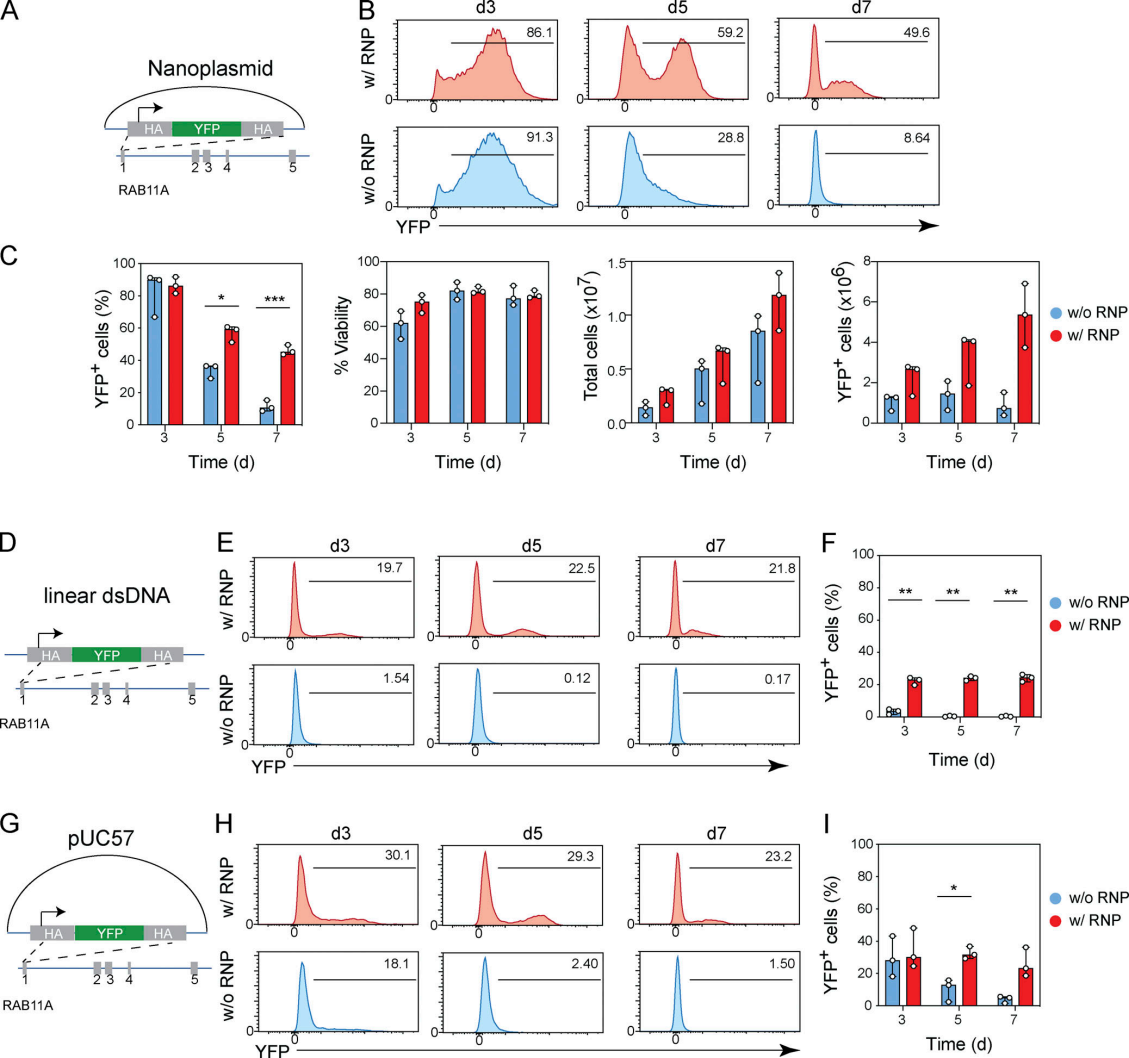
**Kinetics of gene expression following transient transfection of linear dsDNA, plasmid, and nanoplasmid. (A)** Diagram of nanoplasmid knock-in construct *RAB11A*-YFP. **(B and C)** Representative histograms showing the frequencies of CD8^+^ T cells expressing YFP (B) and bar graphs (C) depicting frequency of YFP expression, cell viability, total cell recovery, and edited cell recovery 3, 5, or 7 d after electroporation with promoter-containing nanoplasmid donor template together with (red) or without (blue) Cas9-RNPs targeting the *RAB11A* locus. Circles represent technical replicates; bars represent median values with range (*n* = 3). This experiment was performed twice. **(D)** Diagram of linear dsDNA knock-in construct *RAB11A*-YFP. **(E and F)** Representative histograms showing the frequencies of CD8^+^ T cells expressing YFP (E) and bar graph (F) depicting frequency of YFP expression 3, 5, or 7 d after electroporation with promoter-containing linear dsDNA donor templates together with (red) or without (blue) Cas9-RNPs targeting the *RAB11A* locus. Circles represent technical replicates; bars represent median values with range (*n* = 3). This experiment was performed once. **(G)** Diagram of pUC57 plasmid knock-in construct *RAB11A*-YFP. **(H and I)** Representative histograms showing the frequencies of CD8^+^ T cells expressing YFP (H) and bar graph (I) depicting frequency of YFP expression 3, 5, or 7 d after electroporation with promoter-containing pUC57 plasmid donor templates together with (red) or without (blue) Cas9-RNPs targeting the *RAB11A* locus. Circles represent technical replicates; bars represent median values with range (*n* = 3). This experiment was performed twice. *, P < 0.05; **, P < 0.05; ***, P < 0.001 in Sidak’s multiple comparisons test with RM one-way ANOVA with Geisser–Greenhouse correction.

To assess whether our nonviral CRISPR/Cas9-mediated knock-in approach enabled efficient gene integration beyond the *TRAC* locus, we targeted the *RAB11A* locus using a homology donor construct encoding a YFP-*RAB11A* fusion gene (Roth et al., 2018). As this construct contains the *RAB11A* promoter, YFP expression in transfected T cells could result from either the integrated transgene or from the nonintegrated donor plasmid itself. To identify the right time point for accurately assessing knock-in efficiency (expression of integrated transgene), we transfected CD8^+^ T cells with YFP-*RAB11A* encoding nanoplasmid, pUC57 or linear dsDNA without Cas9-RNP (where YFP expression originates from the non-integrated template only), or together with *RAB11A*-targeting sgRNA Cas9-RNP (where YFP expression could originate from both the non-integrated and integrated template). 3 d after nucleofection, we observed 66.9–91.3% YFP-expressing CD8^+^ T cells in cultures transfected with nanoplasmid donor template alone, and 81.5–91.8% in T cells transfected with nanoplasmid and sg*RAB11A* Cas9-RNP (Fig. S4, B and C). This suggested that, at this time point, YFP-expression derived largely from nonintegrated nanoplasmid. YFP expression was transient and decreased over time to 8.6–15.4% by day 7 after transfection in T cells transfected with nanoplasmid alone. In contrast, YFP expression in T cells transfected with nanoplasmid plus sg*RAB11A* Cas9-RNP stabilized at 43.7–49.6% (Fig. S4, B and C), thus revealing the fraction of T cells with targeted integration of the YFP transgene.

Interestingly, the prolonged transgene expression from nonintegrated promoter-containing donor templates was a unique feature of the nanoplasmid backbone, as electroporation of the construct encoded by linear dsDNA (Fig. S4, D–F) or the usage of a pUC57 plasmid (Fig. S4, G–I) resulted in substantially shorter transient YFP expression. These studies demonstrated that when using promoter-containing nanoplasmid donor templates, the knock-in efficiency measured by transgene expression could only be accurately assessed at least 7 d after electroporation. Moreover, these findings suggested a potential utility of nanoplasmids for broad applications in transient reporter gene expression in T cells.

**Figure 5.**
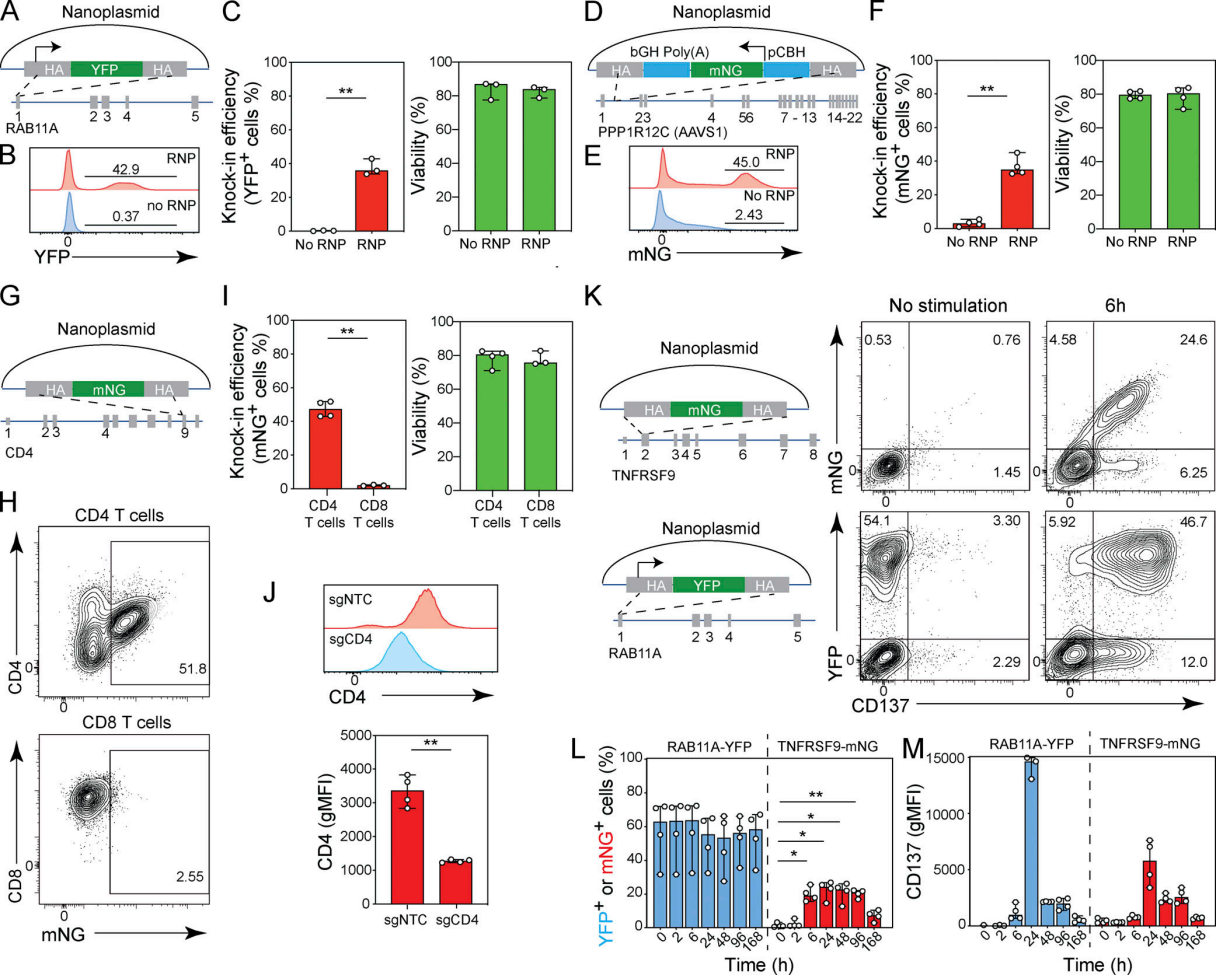
**Generation of reporters of gene expression. (A)** Diagram of nanoplasmid knock-in construct *RAB11A*-YFP. **(B and C)** Histogram overlay for YFP expression (B) and bar graphs (C) showing the frequency of YFP expression and cell viability of CD8^+^ T cells transfected with *RAB11A*-YFP nanoplasmid with or without *RAB11A* targeting Cas9-RNP 10 d after electroporation. Circles represent individual donors; bars represent median values with range (*n* = 3). This experiment was performed three times. **(D)** Diagram of nanoplasmid knock-in construct *AAVS1-*mNG. **(E and F)** Histogram overlay for mNG expression (E) and bar graphs (F) showing the frequency of mNG expression and cell viability of CD8^+^ T cells transfected with *AAVS1-*mNG nanoplasmid with or without *AAVS1* targeting Cas9-RNP 10 d after electroporation. Circles represent individual donors, and bars represent median values with range (*n* = 4). This experiment was performed three times. **(G)** Diagram of nanoplasmid knock-in construct *CD4-*mNG. **(H and I)** Representative contour plots (H) and bar graphs (I) showing the frequency of CD4^+^ and CD8^+^ T cells expressing mNG and cell viability 10 d after electroporation of a nanoplasmid donor template and Cas9-RNP targeting the *CD4* locus. Circles represent individual donors, and bars represent median values with range (*n* = 4 for CD4^+^ T cells, *n* = 3 for CD8^+^ T cells). This experiment was performed twice. **(J)** Histogram overlay for CD4 expression in CD4^+^ T cells transfected with *CD4-*mNG nanoplasmid together with a non-targeting control Cas9-RNP (sgNTC) or a Cas9-RNP targeting the *CD4* locus (sg*CD4*) 10 d after electroporation. **(K)** Diagrams of nanoplasmid knock-in constructs *TNFRSF9*-mNG and *RAB11A*-YFP (left) and representative contour plots (right) showing the frequency of CD8^+^ T cells expressing CD137 and mNG after electroporation with a nanoplasmid mNG reporter construct targeting the *TNFRSF9* locus or a constitutive YFP expressing construct targeting the *RAB11A* locus together with the respective Cas9-RNP either without restimulation or 6 h after restimulation with Transact. **(L)** Bar graphs showing the frequency of YFP (blue) and mNG (red) expressing CD8^+^ T cells over time after electroporation with a nanoplasmid mNG reporter construct targeting the *TNFRSF9* locus or a constitutive YFP expressing construct targeting the *RAB11A* locus together with the respective Cas9-RNP and restimulation with Transact at time 0 h. Circles represent individual donors; bars represent median values with range (*n* = 4). This experiment was performed twice. **(M)** Bar graphs showing the geometric mean fluorescent intensity (gMFI) of CD137 expression in CD8^+^ T cells over time after electroporation with a nanoplasmid mNG reporter construct targeting the *TNFRSF9* locus or a constitutive YFP expressing construct targeting the *RAB11A* locus together with the respective Cas9-RNP and restimulation with Transact at time 0 h (*n* = 4). Circles represent individual donors; bars represent median values with range. *, P < 0.05; **, P < 0.01 in paired *t* test (C, F, I, and J) or in RM one-way ANOVA with Geisser–Greenhouse correction (L).

Based on these initial results, going forward we evaluated knock-in rates using promoter-containing donor templates 10 d after transfection and included a No-RNP control. For the YFP-*RAB11A* construct we thus measured a 33.8–42.9% knock-in rate across three T cell donors, with no detectable expression in the No-RNP condition (Fig. 5, A–C).

We next targeted the *AAVS1* safe harbor locus, located in intron 1 of the *PPP1R12C* (protein phosphatase 1 regulatory subunit 12C) gene. Targeting of this site is not expected to induce adverse physiological effects upon disruption, and allows robust expression of exogenously inserted genes (Smith et al., 2008; Hockemeyer et al., 2009; Chu et al., 2015). We designed a nanoplasmid donor construct (Fig. 5 D) expressing mNG under the control of the chicken/β-actin hybrid intron promoter (Gray et al., 2011), targeting the *AAVS1* locus with 500 bp homology arms. On day 10 after transfection, we observed 32.5–45% knock-in efficiency across four donors tested (Fig. 5, E and F). Taken together, our approach enabled targeting of transgenes to safe harbor loci with high efficiency.

### Generation of gene-expression reporters

We next wanted to demonstrate that our approach could deliver transgenes that faithfully reported the transcriptional activity of endogenous genes. As proof-of-concept, we first targeted the *CD4* locus, which is active in CD4^+^ T cells but inactive in CD8^+^ T cells, with a nanoplasmid donor template designed to create a bicistronic transcript where the existing *CD4* gene is fused in frame at the C terminus with a P2A peptide and mNG (Fig. 5 G). When we transfected CD4^+^ T cells with sg*CD4* Cas9-RNP and nanoplasmid template, we observed 43–51.8% of the T cells concomitantly expressing mNG and CD4, whereas no mNG expression was observed in CD8^+^ T cells (Fig. 5, H and I). Of note, CD4 expression levels in CD4^+^ T cells that had successfully integrated the mNG gene were lower (on average about half) than in control CD4^+^ T cells transfected with a non-targeting control guide RNA (Fig. 5 J), suggesting that in most cells only one *CD4* allele was successfully recombined, whereas the second allele was disrupted, likely resulting in a loss-of-function mutation.

We next attempted to generate a reporter for T cell activation in primary CD8^+^ T cells by targeting the *TNFRSF9* gene, which encodes the activation marker CD137 (Ward-Kavanagh et al., 2016), and is transiently up-regulated following TCR stimulation. We designed a nanoplasmid donor template targeting the first coding exon (exon 2) of the *TNFRSF9* gene and inserted mNG followed by P2A in frame with the N-terminus of CD137, thus generating a CD137 reporter gene (Fig. 5 K). After nucleofection with *TNFRSF9-*mNG nanoplasmid and sg*TNFRSF9* Cas9-RNP, we cultured CD8^+^ T cells for 10 d in order for any CD137 expression stemming from the initial T cell activation to subside before re-stimulating with TransAct. We then measured mNG expression by flow cytometry over the course of 7 d. Control T cells that had been transfected with *RAB11A*-YFP and sg*RAB11A* Cas9-RNP constitutively expressed YFP, irrespective of TCR stimulation, and up-regulated CD137 expression by 6 h following restimulation (Fig. 5, K and L). In contrast, CD8^+^ T cells transfected with the *TNFRSF9*-mNG construct did not express mNG or CD137 without restimulation or 2 h after restimulation, but up-regulated and co-expressed both as early as 6 h after stimulation (Fig. 5, K and L). mNG expression faithfully recapitulated CD137 expression for 7 d (168 h) after TCR stimulation (Fig. 5, L and M), reaching a maximum at 24 h and declining between days 4 and 7 after stimulation. Similar to our observation with the CD4 reporter, we found that the expression level of CD137 itself was reduced by about half in T cells that expressed the mNG reporter (Fig. 5 M), again suggesting that only one allele had incorporated the reporter, whereas the second *TNFRSF9* allele had been disrupted.

Our data demonstrated that knock-in fusion constructs reliably reported transcriptional activity in primary human T cells but may alter target gene expression levels. Improved construct designs, targeting strategies, or transgenic cell selection methods may help to minimize these effects.

### Efficient multiplexed gene knock-in in human T cells

**Figure 6.**
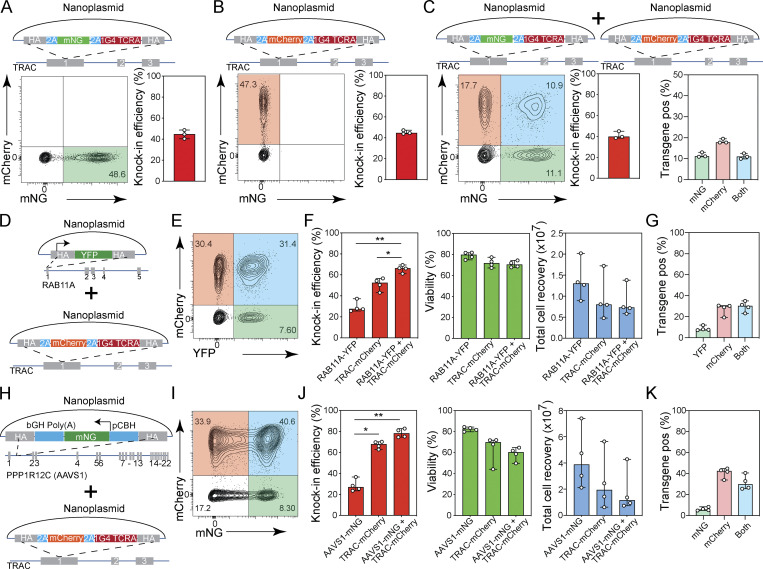
**Multiplexed gene knock-in in human T cells. (A–C)** Diagrams of nanoplasmid knock-in constructs are provided on the top. Representative contour plots (left) and bar graphs (right) showing the frequency of CD8^+^ T cells expressing mNG (A) 10 d after electroporation with a nanoplasmid *TRAC*-mNG donor template and Cas9-RNPs targeting the *TRAC* locus, mCherry (B) 10 d after electroporation with a nanoplasmid *TRAC*-mCherry donor template and Cas9-RNPs targeting the *TRAC* locus, or either mNG or mCherry (C) 10 d after electroporation with two nanoplasmid donor templates (*TRAC*-mNG and *TRAC*-mCherry) and Cas9-RNPs targeting the *TRAC* locus. Graph on the right for C indicates proportion of transgene expressing cells that express mNG (green), mCherry (red), or both (blue). Circles represent individual donors; bars represent median values with range (*n* = 3). This experiment was performed three times. **(D–F)** Diagrams of nanoplasmids used in dual targeting study, *RAB11A*-YFP and *TRAC*-mCherry (D); representative contour plot (E) showing the frequency of CD8^+^ T cells expressing YFP, mCherry, or both; and bar graphs (F) showing knock-in efficiency, cell viability, and total cell recovery of CD8^+^ T cells 10 d after electroporation with nanoplasmid donors *RAB11A*-YFP and *TRAC*-mCherry and Cas9-RNPs targeting the *RAB11A* and *TRAC* loci. **(G)** Proportion of transgene expressing T cells co-transfected with nanoplasmid donors *RAB11A*-YFP and *TRAC*-mCherry and Cas9-RNPs targeting the *RAB11A* and *TRAC* loci that express YFP (green), mCherry (red), or both (blue). Circles represent individual donors, and bars represent median values with range (*n* = 4). This experiment was performed three times. **(H)** Diagrams of nanoplasmids used in dual targeting study, *AAVS1*-mNG and *TRAC*-mCherry. **(I and J)** Representative contour plot showing the frequency of CD8^+^ T cells expressing mNG, mCherry or both (I) and bar graphs (J) showing knock-in efficiency, cell viability, and total cell recovery of CD8^+^ T cells 10 d after electroporation with nanoplasmid donors *AAVS1*-mNG and *TRAC*-mCherry and Cas9-RNPs targeting the *AAVS1* and *TRAC* loci. **(K)** Proportion of transgene expressing cells co-transfected with nanoplasmid donors *AAVS1*-mNG and *TRAC*-mCherry and Cas9-RNPs targeting the *AAVS1* and *TRAC* loci that express mNG (green), mCherry (red), or both (blue). Circles represent individual donors; bars represent median values with range (*n* = 4). This experiment was performed twice. *, P < 0.05; **, P < 0.01 in RM one-way ANOVA with Geisser–Greenhouse correction.

**Figure S5.**
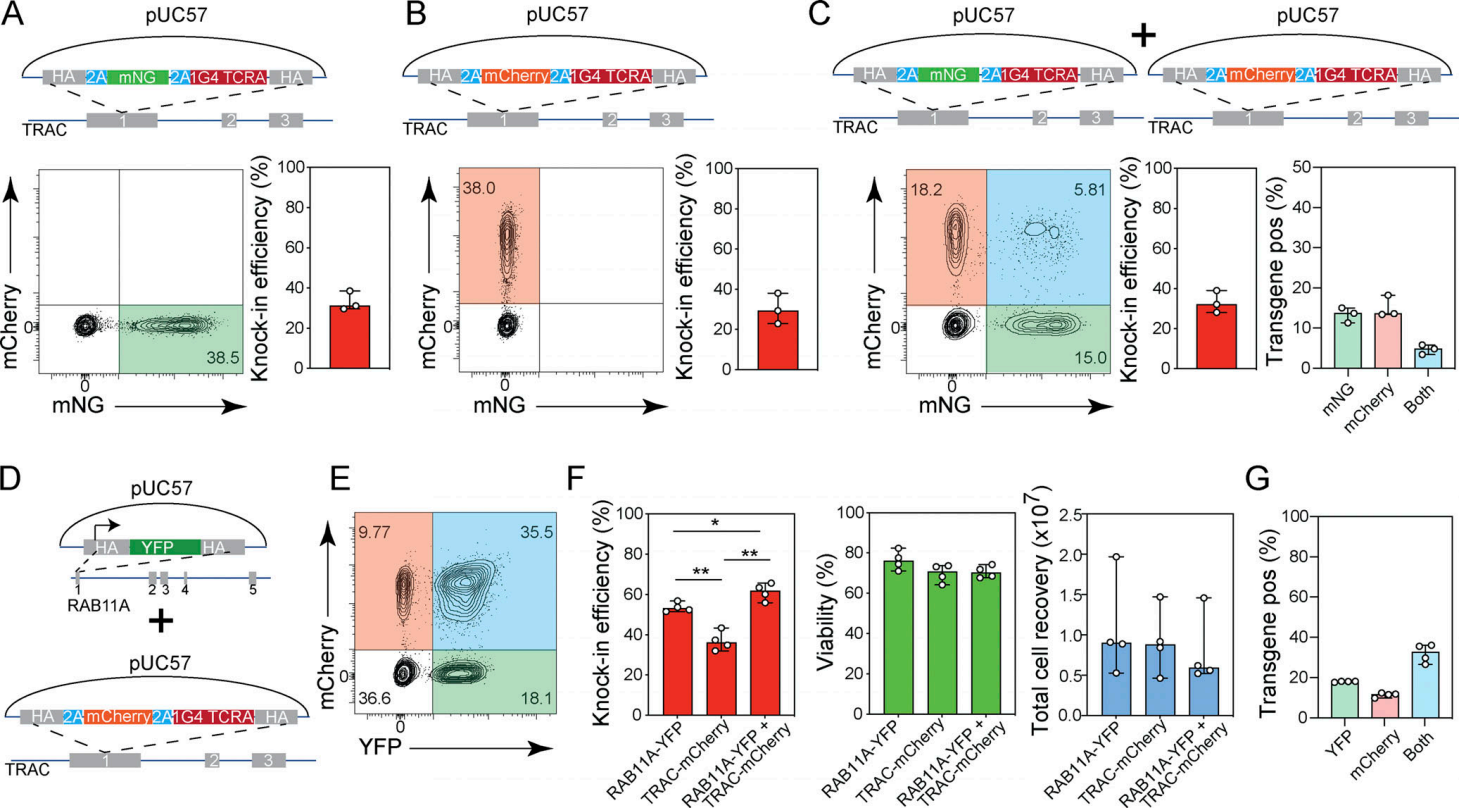
**Multiplexed gene knock-in in human T cells. (A–C)** Diagrams of pUC57 plasmid knock-in constructs are provided on the top. Representative contour plots (left) and bar graphs (right) showing the frequency of CD8^+^ T cells expressing mNG (A) 10 d after electroporation with a pUC57 plasmid *TRAC*-mNG donor template and Cas9-RNPs targeting the *TRAC* locus (*n* = 3), mCherry (B) 10 d after electroporation with a pUC57 plasmid *TRAC*-mCherry donor template and Cas9-RNPs targeting the *TRAC* locus (*n* = 3), or either mNG or mCherry (C) 10 d after electroporation with two pUC57 plasmid donor templates (*TRAC*-mNG and *TRAC*-mCherry) and Cas9-RNPs targeting the *TRAC* locus (*n* = 3). Graph on the right for C indicates proportion of transgene expressing cells that express mNG (green), mCherry (red), or both (blue). Circles represent individual donors; bars represent median values with range. This experiment was performed three times. **(D)** Diagrams of pUC57 plasmids used in dual targeting study, *RAB11A*-YFP and *TRAC*-mCherry. **(E and F)** Representative contour plot showing the frequency of CD8^+^ T cells expressing YFP, mCherry or both (E) and bar graphs (F) showing knock-in efficiency, cell viability, and total cell recovery of CD8^+^ T cells 10 d after electroporation with pUC57 donors *RAB11A*-YFP and *TRAC*-mCherry and Cas9-RNPs targeting the *RAB11A* and *TRAC* loci. **(G)** Proportion of transgene expressing cells co-transfected with pUC57 donor templates *RAB11A*-YFP and *TRAC*-mCherry and Cas9-RNPs targeting the *RAB11A* and *TRAC* loci that express YFP (green), mCherry (red), or both (blue). Circles represent individual donors; bars represent median values with range (*n* = 4). This experiment was performed three times. *, P < 0.05; **, P < 0.01 in RM one-way ANOVA with Geisser–Greenhouse correction.

Given the reduced target gene expression observed with our knock-in reporters, we wanted to further assess the potential for biallelic transgene integration with our approach. In order to do so, CD8^+^ T cells were transfected with a *TRAC*-targeting nanoplasmid carrying either a mNG or a mCherry reporter gene (Fig. 6, A and B) or with both nanoplasmid donors combined in equal quantities (Fig. 6 C). The mNG and mCherry constructs alone resulted in 40.4–48.6% and 43.9–47.3% knock-in efficiency, respectively (Fig. 6, A and B). When both donor templates were co-transfected, the overall knock-in rate remained at 38.5–45% (Fig. 6 C). Of all T cells in culture, 10.9–13% expressed mNG only, 17.7–19.5% expressed mCherry only, and 9.9–12.5% expressed both reporters (Fig. 6 C). This meant that 25.7–27.8% of the cells that successfully integrated the donor template did so on both alleles. Overall knock-in rates were slightly lower when using equivalent pUC57 plasmid templates (22.9–39%), and only 3.5–5.8% of all cells had a detectable biallelic integration (Fig. S5, A–C). Of note, this experimental strategy will underestimate the true rate of biallelic integration, given that the single-positive cell population detected by flow cytometry may bear either one or two copies of the same donor template. The high degree of biallelic integration that we observed at the *TRAC* locus was perhaps surprising, given that only about 10% of T cells express two functional TCR α chains on their surface (Schuldt and Binstadt, 2019). However, allelic exclusion as observed for the TCR β locus does not operate at the TCR α locus, and indeed about 30% of T cells express two functional TCR α mRNAs (Dash et al., 2011; Redmond et al., 2016; Schuldt and Binstadt, 2019). Given that our *TRAC* targeting strategy generates an in-frame fusion with the endogenous *TRAC* gene, the rate of biallelic integration observed here is in line with these prior findings.

Engineering of complex genetic circuits or multiplex reporter assays may require more than one gene edit at different loci for full effectiveness. Therefore, we assessed our protocol for integration of two homology donor templates at distinct genomic loci. We first tested a combination of a nanoplasmid donor containing the YFP-*RAB11A* transgene with a construct encoding mCherry-P2A as an in-frame fusion with the *TRAC* constant region (Fig. 6 D). We transfected CD8^+^ T cells with 2 µg of either construct alone or the combination of both together with the respective sg*RAB11A* and sg*TRAC* Cas9-RNPs and assessed reporter expression by flow cytometry 10 d later. When transfected with YFP-*RAB11A* or *TRAC*-mCherry alone, 25.6–37.3% and 43–56.6% of the T cells expressed the respective reporters (Fig. 6, E and F). Of the cells transfected with both constructs, a total of 61.1–69.4% showed expression of either or both reporters (Fig. 6 F). While 7.3–12% of T cells expressed only YFP and 19.4–30.6% expressed only mCherry, 23.2–35% of all T cells, which corresponded to 38.0–52.7% of transfected cells, co-expressed both transgenes (Fig. 6 G). Although we had doubled the total amount of nanoplasmid (4 µg) for these experiments, we observed only a minor impact on cell viability and recovery (Fig. 6 F), consistent with our initial nanoplasmid titration study. pUC57-based donor templates targeting the same loci yielded comparable dual knock-in rates (Fig. S5, D–G). We obtained similar results when simultaneously targeting the *AAVS1* and *TRAC* loci (Fig. 6, H–K).

These data demonstrated that dual knock-in occurred successfully, without compromising knock-in efficiency and without interference between two distinct constructs.

### Efficient plasmid-based CRISPR/Cas9-mediated gene editing with large payloads

**Figure 7.**
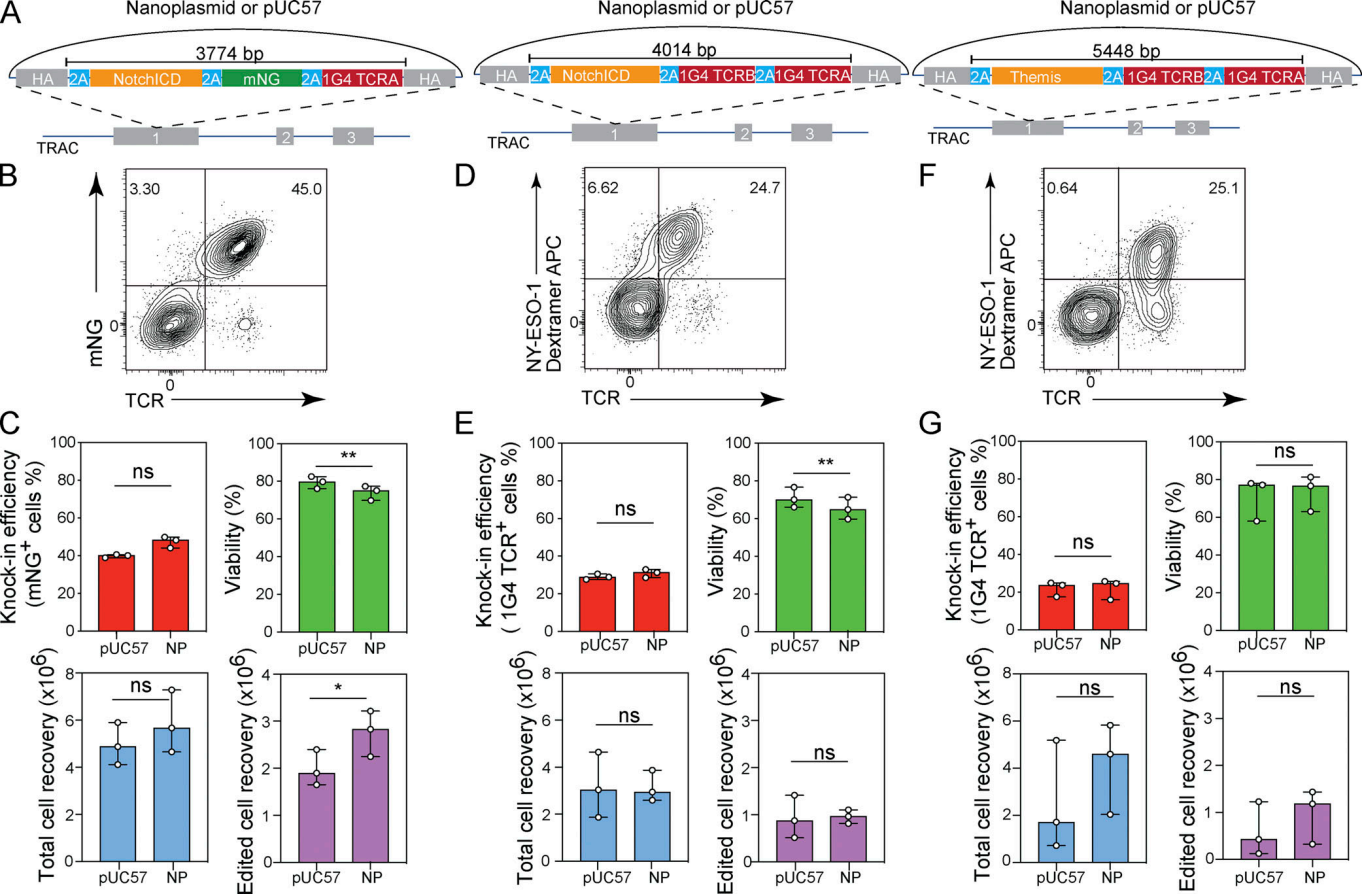
**Nonviral CRISPR gene editing with large payloads. (A)** Diagram of nanoplasmid knock-in constructs *TRAC*_NotchICD_mNG, *TRAC_*NotchICD_1G4, and *TRAC*_THEMIS_1G4. **(B, D, and F)** Representative contour plots showing the frequency of CD8^+^ T cells expressing mNG (B) or 1G4 TCR (D and F) 5 d after electroporation of a NotchICD_mNG (B), NotchICD_1G4 (D), or THEMIS_1G4 (F) nanoplasmid donor template together with Cas9-RNP targeting the *TRAC* locus. **(C, E, and G)** Bar graphs showing the frequency of CD8^+^ T cells expressing mNG (C) or 1G4 TCR (E and G) and cell viability 5 d after electroporation of a NotchICD_mNG (C), NotchICD_1G4 (E), or THEMIS_1G4 (G) nanoplasmid donor template together with Cas9-RNP targeting the *TRAC* locus. Circles represent individual donors, and bars represent median values with range (*n* = 3). This experiment was performed three times. *, P < 0.05; **, P < 0.01 in paired *t* test.

Current gene knock-in approaches do not permit the integration of large DNA sequences. We therefore sought to investigate whether transgenic payloads of greater size, including those that exceed the limitation of AAV-based homology donors, could be integrated using our plasmid-based strategy. The payload (size of the DNA construct excluding homology arms and plasmid backbone) for our 1G4 TCR knock-in construct is 1.5 kb. Using the 1G4 template as the framework, we designed a series of constructs of increasing payload sizes with 0.5-kb homology arms targeting the *TRAC* locus and encoding: the intracellular domain of human Notch1-P2A as an in-frame fusion with mNG-P2A and the 1G4 TCR α chain at 3.8 kb (*TRAC*_NotchICD_mNG); the intracellular domain of Notch1-P2A as an in-frame fusion with the full-length 1G4 TCR at 4 kb (*TRAC*_NotchICD_1G4), and the gene encoding Themis, which plays a regulatory role in both positive and negative T cell selection during late thymocyte development (Fu et al., 2013), as a P2A-in-frame fusion with the full length 1G4 TCR at 5.45 kb (*TRAC*_Themis_1G4, Fig. 7 A). We transfected CD8^+^ T cells with sg*TRAC*/Cas9-RNP and pUC57 or nanoplasmid-based donor templates. For the 3.8-kb *TRAC*_NotchICD_mNG construct, we obtained 38.9–40.6% and 44.1–49.9% knock-in frequencies with the pUC57 and nanoplasmid templates, respectively (Fig. 7, B and C). Transfection of the 4-kb *TRAC*_NotchICD_1G4 construct resulted in 27.1–30.5% (pUC57) and 28.6–32.8% (nanoplasmid) knock-in rates (Fig. 7, D and E). The 5.45-kb *TRAC*_Themis_1G4 donor yielded 17.5–24.9% (pUC57) and 16–25.7% (nanoplasmid) transgene expressing T cells (Fig. 7, F and G). A trend towards higher cell recovery with nanoplasmid DNA donors was observed for two of the three constructs, but did not reach statistical significance (Fig. 7, C, E, and G). Generally, knock-in efficiencies and cell recovery were comparable for the pUC57 and nanoplasmid formats for these larger donor templates, which is likely due to the plasmid backbone accounting for a lower percentage of the overall DNA amount delivered to the T cells.

These data demonstrated that we could increase the payload size to over 5 kb (more than threefold compared to a TCR-encoding construct) and still achieve more than 20% knock-in efficiency with our plasmid-based CRISPR gene editing approach.

## Discussion

Precision gene editing in primary human T cells has the potential to quickly advance our understanding of T cell biology and to be transformative for next generation engineered T cell therapies. Emerging approaches that use DNA nuclease technologies have enabled mutant gene correction, the introduction of entire genes or gene fusions into a desired location, or the manipulation of regulatory elements, none of which are possible with existing retro- or lentivirus–based methodology (Simeonov and Marson, 2019). Several protocols using AAV vectors, together with Cas9 or Cas12a, were developed to introduce CARs or TCRs into the *TRAC* locus of T cells, often demonstrating ≥50% transgene integration through combined Cas9-RNP electroporation and virus infection (Mansilla-Soto et al., 2022; Zhang et al., 2021). AAV-based gene modification facilitates the generation of T cell populations that can recognize and kill desired target cell types without compromised functionality. However, the use of AAV-based homology donors requires cumbersome and expensive virus production methods (Bak et al., 2018), which currently limits its broader application in the research community. Although sufficient for CAR or TCR editing, the cargo size limit of AAV of approximately 4.8 kb also restricts the broader utility for this engineering modality (Salganik et al., 2015).

These limitations have spurred interest in developing nonviral precision gene editing methods for increased versatility and ease of use, faster turnaround times, and reduced cost. The first breakthrough in this regard was the demonstration that linear dsDNA donor templates, previously thought to be too toxic for T cells, could be used successfully to introduce longer DNA segments, including TCRs (Roth et al., 2018). However, the achieved knock-in efficiencies when using linear dsDNA donors were modest for TCR editing, relative to AAV-based methods. While our study demonstrated that efficiency of gene editing with linear dsDNA could be improved over previously reported methods, the need for production and purification of linear dsDNA in the large quantities required for editing limits the versatility and scalability of the approach.

Our fully nonviral gene editing protocol uses readily available plasmid-based donor templates co-delivered with high-fidelity Cas9-RNPs into primary human T cell populations via electroporation. Through this approach, we achieved knock-in efficiencies at multiple loci (alone or in combination) on par with AAV-based methods and preserved coincident high knock-out efficiency under multiplex editing conditions, thus realizing the full potential of nonviral editing technology regarding broad application, turnaround time, and cost savings. Plasmid donors can be designed and synthesized quickly, inexpensively and with high purity. They offer the opportunity for sequence verification and are amenable to large-scale, good manufacturing practices grade qualification for use in cellular therapies. Importantly, by way of plasmid donors, we demonstrate the successful delivery of genetic cargo >5 kb without a steep drop-off in knock-in efficiency compared to smaller transgenes, suggesting that the delivery of even larger constructs may be possible. Further, under optimal parameters, the introduction of plasmid DNA had minimal impact on cell viability and, more importantly, the recovery of edited cells was similar to loss-of-function perturbations using Cas9-RNP alone.

We do note expected liabilities for all approaches that rely on the creation of dsDNA breaks and/or delivery of foreign genetic material to facilitate HDR. As we and others have demonstrated, chromosomal translocations are a predicted byproduct of multiplex gene perturbation (Stadtmauer et al., 2020). Careful monitoring of such events, regardless of the edited loci, is critical for assessing safety risks in a therapeutic setting, or to understand confounding gene expression changes that might influence T cell functionality after editing. Innate immune sensing (Schlee and Hartmann, 2016) of homology donor DNA and/or AAV capsids (Ronzitti et al., 2020), as well as a DNA damage response triggered by DNA break formation (Nambiar et al., 2022), could stress the modified T cell population. However, these effects may be short-lived as T cells engineered by our approach and similar viral or nonviral methods maintain potent effector function and in vitro expansion potential.

Broadly, our method has the potential to serve as a foundation for unleashing the full potential of precision gene editing in primary human T cells for basic research and clinical applications alike.

## Materials and methods

### Buffy coats

Buffy coats from healthy donors were collected as part of the Genentech blood donor program, with written informed consent and approval from the Western Institutional Review Board.

### Antibodies

All antibodies used for flow cytometric analyses are listed in Table S1.

### Guide RNAs

Where applicable, *Streptococcus pyogenes* Cas9-based targeting sequences (20mers) were identified using a custom sgRNA design tool. Guide RNAs were selected based on their predicted target specificity using the cutting frequency determination specificity score as an off-target specificity prediction algorithm (Doench et al., 2016), as well as two on-target cutting efficiency scores: the Azimuth algorithm, a version of the popular Rule Set 2 on-target cutting efficiency prediction algorithm (Doench et al., 2016), and the DeepCas9 algorithm (Wang et al., 2019). Guide RNAs targeting the *TRAC* and *TRBC* loci were previously described (Roth et al., 2018). All sgRNA sequences are listed in Table S2. All guide RNAs were ordered as Alt-R CRISPR-Cas9 sgRNAs from Integrated DNA Technologies (IDT).

### HDR donor template design

Donor templates were designed in SnapGene (GSL Biotech). To design long HDR templates, the Cas9 cut site of an experimentally validated guide RNA in the vicinity of the desired knock-in site is identified within the genome (3 nt upstream of the protospacer adjacent motif [PAM]) and ∼0.5-kb regions 5′- and 3′- of the site are designated as left and right homology arms, respectively. Any native sequence between the actual guide RNA cut site and the desired knock-in site was included as part of the donor construct between the homology arms to avoid any offset, to ensure perfect binding of the homology arms to the genomic sequence up to the cut site, and to avoid nucleotide sequence duplications, this region should be codon-optimized. The sequence of any cargo is then included in the construct in frame with the target locus, if so desired. If not required for other reasons, codon optimization should be avoided, as it can reduce knock-in efficiency or impact transgene expression relative to an endogenous equivalent. Guide RNA binding sites within the donor template need to be mutated as extensively as possible (preferably mutation of the PAM, followed by maximum mutations within the spacer binding site). If using an existing gene transcript to express an exogenous protein, the cut site should be located within the coding sequence of the target gene. A GSG-2A site is placed downstream of the left homology arm in frame with the target gene, followed by the open reading frame of the exogenous gene. Multiple GSG-2A-Gene cassettes can then be added after the first. Stop codons are excluded from all genes where ribosomal read-through to the next cassette is desired. At the end of the last gene in the series, but before the right homology arm, a stop codon may be inserted, or another GSG-2A site, or a stop codon plus a polyadenylation sequence. Alternatively, the exogenous coding sequence may continue into the right homology arm, to create an in-frame fusion with the target locus.

When designing templates targeting a noncoding region of the genome, left and right homology arms are selected as described above. Between the homology arms are placed an enhancer, promoter, and Kozak sequence, followed by the genes of interest separated by GSG-2A sequences, as necessary. The last gene in the series terminates in a stop codon and polyadenylation sequence. Construct organization is shown, where LHA is the left homology arm (500 bp unless otherwise indicated), GSG is a glycine-serine-glycine linker, T2A and P2A are ribosomal cleavage sequences, furin is an arginine-alanine-lysine-arginine endoprotease cleavage site, bGHpA is the polyadenylation site from the bovine growth hormone gene, pCBH is a transcriptional regulatory element consisting of the CMV enhancer and chicken β-actin promoter, and RHA is right homology arm (500 bp unless otherwise indicated). tCTS sites are truncated Cas9 targeting sequences with PAM sites that bear 4-bp mismatches at the 5′ end. Templates with tCTS sites bear one at the 5′ and 3′ end, both oriented inward and flanked by a 16-bp edge sequence (Nguyen et al., 2019). Donor template sequences are given in Table S3.

### HDR template production

Nanoplasmid and pUC57 HDR templates were provided as primary cell transfection grade material and supplied at a concentration of 1 mg/ml resuspended in water by Nature Technology. *TRAC*_1G4_500HA and *TRAC*_mNG_500HA linear dsDNA donor DNAs were made via PCR (Roth et al., 2018). PCR product was generated using Q5 High-Fidelity Polymerase (NEB) with 0.25 µM forward (5′-AAC​ATA​CCA​TAA​ACC​TCC​CAT​TCT​G-3′) and reverse (5′-TTG​GAG​AGA​CTG​AGG​CTG​GGC​CAC​G-3′) primers and 10 ng/ml plasmid DNA template per reaction. The cycling parameters were 98°C for 15 s, 60°C for 15 s, and 72°C for 1 min, for a total of 30 cycles. The products from 96 × 100-µl reactions were pooled and equilibrated in Qiagen buffer and then purified through a HiSpeed Plasmid Maxi Kit (Qiagen). The final product was eluted in nuclease-free water, and DNA concentration was adjusted to 1 mg/ml.

### Isolation and culture of primary human T cells

Primary human CD8^+^ and CD4^+^ T cells were isolated by positive selection from buffy coats using the StraightFrom Buffy Coat CD8 MicroBead Kit or CD4 MicroBead Kit, respectively, according to the manufacturer’s instructions (Miltenyi Biotec). Residual red blood cells were lysed before culture. Cells were plated at an initial concentration of 1 million cells/ml of stimulation medium. Unless otherwise noted, stimulation medium consisted of PRIME-XV T Cell CDM media (Irvine Scientific) supplemented with IL-7 (Miltenyi Biotec) at 25 ng/ml and IL-15 (Miltenyi Biotec) at 50 ng/ml for CD8^+^ T cells, and IL-7 (25 ng/ml), IL-15 (50 ng/ml), and IL-2 (400 U/ml; BioLegend) for CD4^+^ T cells. T Cell TransAct (Miltenyi Biotec) was added to the cultures at a 1:100 dilution. T cell medium was prepared using the following ingredients: RPMI 1640 (11875093; Gibco), 10% FBS (SH30071.03; Hyclone), 2 mM L-alanyl-L-glutamine (GlutaMAX; Gibco), 1 mM sodium pyruvate (Gibco), 0.1 mM nonessential amino acids (Gibco), 55 μM 2-mercaptoethanol (Gibco), 100 U/ml penicillin, 100 μg/ml streptomycin (PenStrep; Gibco), and 10 mM Hepes (Gibco). Medium was sterilized through a 0.22-μm filter. Unless indicated otherwise, T cells were cultured for 36–48 h before electroporation. The culture volume was expanded to maintain cells at ∼1 million cells/ml over the course of the culture.

### RNP assembly

RNPs were produced by combining target-specific sgRNAs (IDT) and recombinant Cas9 (SpyFi; Aldevron). Briefly, lyophilized sgRNAs were reconstituted in Nuclease-free Duplex Buffer (IDT) to a concentration of 200 µM. For every 60 pmol of Cas9 used, 180 pmol of sgRNA was added to obtain a 3:1 sgRNA:Cas9 ratio. The sgRNA:Cas9 mixture was incubated at room temperature for 15 min to allow RNP formation. For combined TCR knock-in/*TRBC* knock-out experiments, 30 pmol each of *TRAC* and *TRBC* Cas9-RNPs were assembled separately and then mixed together using equal volumes. A total of 60 pmol of combined *TRAC* and *TRBC* Cas9-RNPs were used for a single nucleofection reaction. For knock-in experiments targeting other loci, 60 pmol of total Cas9-RNP was used per nucleofection reaction.

### Nucleofection

After 36–48 h of stimulation, T cells were pelleted, washed with PBS, and gently resuspended in P3 buffer with supplement (Lonza Bioscience) at 2 million cells per 20 μl. The following components of a single nucleofection reaction were added to a PCR tube and mixed gently: preformed RNPs (60 pmol total), HDR template (≤8 µg), and T cells resuspended in P3 buffer. In some cases, poly-L-glutamic acid (Sigma-Aldrich; 150 µg) was also added to the mixture. This mixture was then transferred to 1 well of a 16-well 4D-Nucleofector cuvette (Lonza Bioscience) and pulsed with code EH115 unless otherwise indicated. After electroporation, the 4D-Nucleofector cuvette was placed in a 37°C tissue culture incubator for 15 min to allow for cell recovery. After recovery, the cells were transferred to a 24-well tissue culture plate containing 2 ml of prewarmed PRIME-XV medium supplemented with 25 ng/ml IL-7 and 50 ng/ml IL-15 (CD8^+^ T cells) or 25 ng/ml IL-7, 50 ng/ml IL-15, and 400 U/ml IL-2 (CD4^+^ T cells).

### Flow cytometry

Transfected cells at different time points were analyzed by flow cytometry to measure the knock-in efficacy. All reagents were used according to manufacturer’s recommendations. Briefly, cells were pelleted, washed with PBS, and gently resuspended and incubated for 10 min at room temperature in prediluted Fixable Viability Dye eFluor 780 or propidium iodide. After incubation, cells were washed twice in FACS buffer and subjected to surface staining with fluorochrome-conjugated CD3 and/or anti-TCRα/β, along with anti-CD4 (for CD4^+^ T cells) or anti-CD8 (for CD8^+^ T cells). In some experiments, cells were also stained with either NY-ESO-1_157–165_ or pp65_495–503_ pMHC dextramer (PE or APC) for 10 min at room temperature and protected from light, before surface antibodies were added. After the addition of other surface antibodies, cells were incubated at 4°C in the dark for an additional 15 min. For CD19 CAR staining, cells were first stained with biotin anti-human CD19 CAR detection reagent (Miltenyi Biotec) followed by Streptavidin PE. For staining cells with anti-CD137 PE, a Fluorescence Amplification by Sequential Employment of Reagents Kit – PE (Miltenyi Biotec) was used to amplify the fluorescence intensity. Stained cells were washed twice in FACS buffer before proceeding to FACS acquisition. To calculate the absolute number of cells, CountBright Absolute Count Beads (Thermo Fisher Scientific) were added before FACS acquisition. Samples were acquired using a FACSymphony or an LSR Fortessa equipped with FACSDiva software (all from BD Biosciences). Compensation was performed using single-stained controls prepared with Ultra-comp ebeads (Thermo Fisher Scientific). Flow cytometry standard 3.0 files were imported and analyzed using FlowJo software v3.0 (FlowJo). A conventional gating strategy was used to remove aggregates, and dead cells were excluded based on viability dye staining.

### Simoa assay

IFN-α analysis in pre- and post-electroporation culture supernatants were analyzed using the Simoa IFN-α Advantage Kit (HD-1/HD-X; 100860) according to the manufacturer’s protocol. Briefly, 200 µl of IFN-α calibrators and experimental samples were added to wells in a 96-well plate. The kit’s bead reagent, detector reagent, SBG (streptavidin β galactosidase) reagent, and sample diluent were added to the reagent bay in the Quanterix HD-X, and resorufin-D-galactopyranoside was added to the sample bay. After IFN-α assay setup in Simoa software, the plate containing calibrator and experimental samples was loaded into the sample bay and analyzed on the Quanterix HD-X.

### T cell activation

T cell activation cultures comprised CRISPR-engineered T cells, a HLA-A*02:01^+^ target cell line, and a nontarget HLA-A*02:01-negative target cell line that served as a reference population for the calculation of target cell lysis. Both cell lines were obtained from the Fred Hutchinson International Histocompatibility Working Group. The target and reference cell lines were labeled with CFSE and Cell Trace Violet (CTV; Invitrogen), respectively, to distinguish populations during flow cytometric analysis. For peptide pulsing, CFSE-labeled HLA-A*02:01^+^ target cells were incubated with varying concentrations of the appropriate target peptide at 37°C for 2 h. After the incubation period, cells were washed twice with PBS and then resuspended in 10% FBS RPMI T cell medium. Peptide-loaded CFSE-labeled target cells were cultured with CTV-labeled reference population at a 1:1 ratio, and CRISPR-engineered T cells were added at a 1:1 ratio of T cells to CFSE-labeled target cells. No peptide-added conditions were included as controls. Approximately 24 h later, T cell activation was analyzed as follows: (1) cells were collected and analyzed by flow cytometry to determine CD137 (clone 4B4-1; BioLegend) upregulation and target cell lysis, and (2) supernatants were collected for analysis of effector molecule production by Luminex. For analysis of target cell lysis, CountBright Absolute Count Beads (Thermo Fisher Scientific) were added to flow cytometric analysis samples to quantify the numbers of CFSE-labeled target cells and CTV-labeled nontarget cells during FACS acquisition. Specific target cell lysis was calculated using the following equation: percentage specific lysis = [1 − (no-peptide control ratio/experimental ratio)] × 100. Ratios were calculated by dividing the numbers of the CTV-labeled reference population by the numbers of CFSE-labeled HLA-A*02:01^+^ target cells.

### In vitro killing assay

The A-375 (malignant human melanoma) cells that express the NY-ESO-1 antigen were labeled with 1 µM of Incucyte Cytolight Rapid Dyes (4706) and plated in a 96-well plate at the seeding density of 50,000 cells. 2 h after seeding, a caspase-3/7 green apoptosis reagent (2272582; Invitrogen) and IG4 TCR KI or KO controls (50,000 cells per well) were added to A-375 cells. Cell killing was measured by evaluating the number of A375 cells present in each well expressing caspase-3/7 reagent. The co-culture was monitored for growth and apoptosis using the IncuCyte imaging system for 18 h. After co-culturing, CD137 expression on CD8^+^ T cells was measured by Flow Cytometry (clone 4B4-1; BioLegend).

### Activation of CD4^+^ T cells with CD19 CAR construct

50,000 CD4^+^ T cells with a CD19-specific CAR or a pp65-specific 6-2 TCR (control irrelevant TCR) were plated at a 1:1 E:T ratio with CD19 expressing Granta-519 B cells and incubated for 24 h. Culture supernatants were analyzed for IFN-γ and TNF-α production by Luminex.

### T cell expansion cultures/lactate measurement

Activated CD8^+^ T cells were electroporated at 48 h of culture with only the sg*TRAC* and sg*TRBC* RNPs (knock-out) or with sg*TRAC* RNP, sg*TRBC* RNP, and the TCR-encoding nanoplasmid (knock-in). As a control for no electroporation (no RNP), CD8^+^ T cells were added to the Lonza electroporation cuvette but not subjected to an electroporation pulse code. The no-RNP, knock-out, and knock-in T cells were cultured in a 24-well G-Rex plate (Wilson-Wolf) in PRIME-XV medium supplemented with 25 ng/ml IL-7 (Miltenyi) and 50 ng/ml IL-15 (Miltenyi). Supernatants were collected from the no RNP, knock-out, and knock-in conditions on day 1 after electroporation and every 2–3 d thereafter for 7 d.

Extracellular lactate levels were analyzed as a surrogate for cell proliferation (Grist et al., 2018) using the Lactate-Glo Assay (Promega) according to the manufacturer’s protocol. Briefly, after sample thaw, the Luciferin Detection Solution was brought to room temperature and all other kit components were maintained on ice. Lactate dehydrogenase was reconstituted using water and then placed on ice. Immediately before use, the Lactate Detection Reagent was prepared by mixing the Luciferin Detection Solution, Reductase, Reductase Substrate, Lactate Dehydrogenase, and NAD at ratios specified by the manufacturer. Cell culture supernatants were diluted in PBS, and 50 μl samples or lactate control was added to a 96-well plate followed by 50 μl Lactate Detection Reagent. The plate was shaken for 30–60 s and incubated for 60 min at room temperature. Luminescence was recorded using a plate-reading luminometer.

### Translocation assay

A set of ddPCR-based assays were developed to detect potential chromosomal translocations during simultaneous CRISPR-mediated editing of the three target sites (*TRAC*, *TRBC1*, and *TRBC2*) in engineered T cells (Bio-Rad’s QX200 ddPCR platform). These six translocations were designated as *TRAC*-*TRBC1*, *TRAC*-*TRBC2*, *TRBC1*-*TRAC*, *TRBC1*-*TRBC2*, *TRBC2*-*TRAC*, and *TRBC2*-*TRBC1*. A reference assay to detect the *RPP30* gene of interest was used to measure the ratio of target sequence (copies/μl) over the *RPP30* sequence as the measure of chromosomal translocation at each DNA target site. Primer and probe sequences are: *TRAC* forward, 5′-TGG​GGC​AAA​GAG​GGA​AAT​GAG-3′, *TRAC* reverse, 5′-AGA​ACC​TGG​CCA​TTC​CTG​AAG-3′; *TRAC*-Probe, 5′-CAT​GTG​CAA​ACG​CCT​TCA​ACA​ACA​G-3′; *TRBC1*, 5′-CTG​GGA​TGG​TGA​CCC​CAA​AA-3′; *TRBC1* reverse, 5′-GGC​CAC​ATA​GAA​AGG​GGA​CC-3′; *TRBC1*, probe, 5′-ACC​ATG​AAG​GAG​AAT​TGG​GCA​CCT-3′; *TRBC2* forward, 5′-GGG​GGA​TGG​ACA​GAC​AAT​GG-3′; *TRBC2* reverse, 5′-GCT​GAC​CCT​GTG​AAC​CTT​GA-3′; *TRBC2*-Probe: 5′-ATC​CAG​GTA​GCG​GAC​AAG​ACT​AGA​T-3′; *RPP30* forward, 5′-TCA​GCC​ATA​TTG​TCC​CCT​AAA​CT-3′; *RPP30* reverse, 5′-TGG​TCT​GTC​CAT​GGC​ATC​TT-3′; and *RPP30* probe, 5′-CTG​TAT​GGA​CAC​AGT​GCC​TA-3′.

Genomic DNA isolated from T cells transfected with sg*TRAC* and sg*TRBC* Cas9-RNPs together with a *TRAC* targeting 1G4 TCR-encoding nanoplasmid donor was examined by these ddPCR assays, and translocations were reported as % ratio relative to the reference assay.

### RNA-seq analysis

Human CD8^+^ T cells were isolated from five donors and activated as indicated above followed by electroporation with 60 pmol *TRAC* Cas9-RNP, without or with 3 μg of *TRAC*-mNG template (500 bp homology arms) in either linear dsDNA (PCR) or nanoplasmid format. 20 h after electroporation, RNA was isolated from the cells using an RNeasy Mini kit (Qiagen) according to the manufacturer’s instructions with an on-column deoxyribonuclease (DNase) I digestion. Differential expression analysis of the transcriptome data was performed using the R package DESeq2 (Anders and Huber, 2010). Heatmaps were generated by transforming RNA-seq reads count into normalized expression using variance stabilizing transformation. Gene set enrichment analysis (GSEA) was performed using R Bioconductor package enrichplot (Yu, 2021) and msigdbr (Subramanian et al., 2005; Liberzon et al., 2015). MSigDB Hallmark 2020 gene sets were used for GSEA analysis.

### Statistical analysis

GraphPad Prism software was used for plotting graphs and statistical analysis. Paired *t* test or repeated-measures (RM) one-way ANOVA with Geisser–Greenhouse correction were used to determine statistical significance unless indicated otherwise.

### Online supplemental material

Fig. S1 shows titration of linear dsDNA and nanoplasmid donor templates in RPMI/FBS medium and knock-in of linear dsDNA or nanoplasmid donor templates with PGA and tCTS. Fig. S2 shows cytokine and RNA sequencing data describing the stress response following exposure to dsDNA donor templates. Fig. S3 shows data on TCR editing efficiency in CD4^+^ T cells, quantification of translocation events during TCR editing as well as T cell activation, cytokine production, and target cell killing following TCR editing. Fig. S4 shows kinetics of gene expression following transient transfection of dsDNA, plasmid and nanoplasmid. Fig. S5 shows efficiency of multiplexed gene knock-in in human T cells using pUC57 donor templates. Table S1 lists antibodies used for flow cytometric analyses. Table S2 lists sequences of sgRNAs. Table S3 lists donor template constructs.

## Supplementary Material

Table S1 lists antibodies used for flow cytometric analyses.

Table S2 lists sequences of sgRNAs.

Table S3 lists donor template constructs.

## Data Availability

Sequencing data were deposited in the European Genome-phenome Archive, which is hosted by the European Bioinformatics Institute, under accession no. EGAS00001006125.
